# TS-SSA: An improved two-stage sparrow search algorithm for large-scale many-objective optimization problems

**DOI:** 10.1371/journal.pone.0314584

**Published:** 2025-03-17

**Authors:** Xiaozhi Du, Kai Chen, Hongyuan Du, Zongbin Qiao

**Affiliations:** School of Software Engineering, Xi’an Jiaotong University, Shaanxi, China; Huazhong University of Science and Technology, CHINA

## Abstract

Large-scale many-objective optimization problems (LSMaOPs) are a current research hotspot. However, since LSMaOPs involves a large number of variables and objectives, state-of-the-art methods face a huge search space, which is difficult to be explored comprehensively. This paper proposes an improved sparrow search algorithm (SSA) that manages convergence and diversity separately for solving LSMaOPs, called two-stage sparrow search algorithm (TS-SSA). In the first stage of TS-SSA, this paper proposes a many-objective sparrow search algorithm (MaOSSA) to mainly manages the convergence through the adaptive population dividing strategy and the random bootstrap search strategy. In the second stage of TS-SSA, this paper proposes a dynamic multi-population search strategy to mainly manage the diversity of the population through the dynamic population dividing strategy and the multi-population search strategy. TS-SSA has been experimentally compared with 10 state-of-the-art MOEAs on DTLZ and LSMOP benchmark test problems with 3-20 objectives and 300-2000 decision variables. The results show that TS-SSA has significant performance and efficiency advantages in solving LSMaOPs. In addition, we apply TS-SSA to a real case (automatic test scenarios generation), and the result shows that TS-SSA outperforms other algorithms on diversity.

## Introduction

With the rapid development of technology in various industries, a large number of optimization problems of high complexity have emerged. These optimization problems may involve high-dimensional decision variables, complex nonlinear constraints, expensive computational evaluation of objective functions, and the need to optimize multiple conflicting objectives simultaneously [1,2]. The most common of them are multi-objective optimization problems (MOPs). Some studies have achieved good results by converting MOPs into single-objective problems through the weighting method. However, in the weighting method, the weight value of each objective is predetermined, which leads to the difficulty of setting the optimal weight values when there are complex coupling relationships between multiple objectives [3]. To better solve MOPs, researchers have proposed a large number of multi-objective optimization algorithms. Among them, the performance of multi-objective evolutionary algorithms (MOEAs) is particularly outstanding [4–9]. These traditional MOEAs perform better in solving two-objective and three-objective optimization problems, but their performance decreases as the number of objectives increases [10]. And in real-world applications, the optimization problems often involve more than three objectives, which are called many-objective optimization problems (MaOPs). Many-objective optimization algorithms are an important area of research in optimization today [11]. In addition, some MaOPs involve high-dimensional decision variables, and these problems are also called large-scale many-objective optimization problems (LSMaOPs). LSMaOPs have a more complex search space, which poses greater challenges to the performance of optimization algorithms. In the past few years, many excellent many-objective optimization evolutionary algorithms (MaOEAs) have been proposed [12–16], which have achieved good results in solving MaOPs. But their performance on LSMaOPs is poor because these algorithms mainly consider high-dimensional objectives and ignore high-dimensional decision variables. Some of other studies have proposed large-scale optimization algorithms [17–19], but they mainly solve large-scale multi-objective problems (LSMOPs). Only a few studies have considered both many objectives and high-dimensional decision variables [20,21]. The main challenge of LSMaOPs is to maintain the convergence and diversity of the population in a huge search space. However, most MaOEAs use a single search strategy, which lead to poor performance of these methods under huge search spaces. Genetic algorithms (GA) and differential evolutionary algorithms (DE) are the most commonly used search operators in MaOEAs. However, a large number of studies have shown that biological population intelligence algorithms [22–24] have significantly better performance than GA and DE on single-objective optimization problems. But few studies have been conducted to extend such algorithms to MaOPs. To solve the above problems, this paper proposes a large-scale many-objective evolutionary algorithm (LSMaOEA) based on the sparrow search algorithm, termed the two-stage sparrow search algorithm (TS-SSA). The main contributions of this paper are as follows:

1. This paper proposes the TS-SSA for solving LSMaOPs. TS-SSA is divided into two stages to manage the convergence and diversity of the population separately. The first stage mainly manages convergence and the second stage mainly manages diversity of the population. The two stages will adaptively alternate according to the population characteristics, thus balancing the convergence and diversity of the algorithm.
2. Based on SSA, this paper proposes an adaptive population dividing strategy and a random bootstrap search strategy, which effectively improve the convergence of the algorithm and enable the SSA to be applied to LSMaOPs for the first time.
3. Based on the internal mechanism of the SSA, this paper proposes a dynamic multi-population search strategy to mainly manage the diversity of the population through the dynamic population dividing strategy and the multi-population search strategy, which enables full exploration of large-scale decision spaces to avoid trapping in local optimal and increase population diversity.

The rest of this paper is organized as follows. Sect 2 reviews related work and discusses the unresolved issues. In Sect 3, the proposed TS-SSA method is described in detail. In Sect 4, some experiments are conducted to evaluate the feasibility and effectiveness of our method. Sect 5 concludes this paper and discusses some future work.

## Related work

### Many-objective optimization evolutionary algorithms

Researchers have proposed many MaOEAs in the past few years. These algorithms can be classified into four categories: selection pressure-based, decomposition-based, metrics-based, and dimensionality reduction-based.

In MaOPs, solutions are in a non-dominant relationship with each other in most cases, which is also known as the dominance resistance. In contrast, the selection pressure based on the Pareto dominance is not sufficient to discriminate between solutions, which makes it difficult for the traditional MOEAs to converge [25]. He et al. [13] constructed a fuzzy Pareto dominance relation based on fuzzy logic to increase the selection pressure. Yuan et al. [14] introduced a *α* = 0 . 10-dominance relation in the reference point selection strategy, which divides the solution set into multiple groups based on the reference points, and maintains a competitive relationship within each group. This method increases the selection pressure by reducing the number of solutions involved in the selection. On the other hand, Zhang et al. [15] introduced inflection point driving to improve the convergence of the algorithm. There are also studies that increase the selection pressure by improving the environment selection strategy [12] or proposing a new dominance relation [26]. Most of these methods improve the convergence of the algorithms from the perspective of selecting the offspring with higher convergence. In LSMaOPs, due to the many objectives and high-dimensional decision variables, there is a greater computational burden of complex dominance relationships, which makes it difficult for the algorithms to maintain good performance.

Decomposition-based methods are based on the idea of divide and conquer, where complex MaOPs are decomposed into some smaller MOPs or single-objective problems to be solved. MOEA/D, proposed by Zhang and Li [27], is a classical decomposition-based method, which improves the convergence and diversity of the method by introducing weight vectors to decompose the MOPs into smaller subproblems to improve the convergence and diversity of the method, which allows MOEA/D to maintain a better performance while possessing a lower computational complexity. Since then, a large number of studies have also made improvements to MOEA/D [28–30]. Among them, MOEA/D-M2M, proposed by Liu et al. [31], used direction vectors instead of weight vectors in MOEA/D. MOEA/D-M2M divides the objective space into multiple subspaces by direction vectors, which realizes the parallel evolution of multiple populations to effectively improve the diversity of the populations. And it solves the problem that it is difficult to achieve the optimal selection of the weight vector values. In addition, SPEA/R [16] decomposed the original objective space based on reference vectors. On the other hand, Asafuddoula et al. [5] introduced reference points in MOEA/D to improve the performance of MOEA/D in solving MaOPs.

The metrics-based methods use performance metrics as selection criteria in the environmental selection strategy. Rostami and Neri [32] proposed a fast search algorithm based on hypervolume (HV) and used Monte Carlo simulation to accelerate the computation of HV. Li et al. [33] proposed a two-stage evolutionary algorithm based on R2 metrics for the initial selection of solutions by R2 metrics, while Sun et al. [34] used inverse generation distance (IGD) for the optimal selection of offspring. IGD has lower computational complexity compared to HV, which results in better performance of the method. Although metrics-based methods show good performance, they still have many drawbacks: First, the computational complexity of most metrics is high. Even HV calculated by Monte Carlo simulation and IGD still have high computational cost. Second, the metrics-based methods rely heavily on the true Pareto front and the shape of the front of the problem to be solved, which leads to the poor results of this type of methods in real-world applications [35,36]. Especially in LSMaOPs, computing performance metrics in each iteration incurs high computational costs.

The dimensionality reduction-based methods are achieved by analyzing the features of the objectives and merging the objectives with the same features to reduce the dimensionality of the target. Huang et al. [37] improved the ability of MOEA/D to solve constrained problems by introducing principal component analysis to MOEA/D. S. Liu et al. [38] introduced an adaptive clustering method to cluster the population, which allowed the populations to better fit the Pareto front. R. Liu et al. [39] improved the performance of the method in dealing with LSMOPs by clustering the decision variables with dimensionality reduction, which divided the decision variables into convergence and diversity variables, and treated the two groups of variables separately.

The above methods employ different strategies from different perspectives to improve the convergence and diversity of the population. Selection pressure-based methods and metrics-based methods mainly improve the environmental selection strategy to increase the selection pressure. However, in LSMaOPs, many objectives and high-dimensional decision variables make it difficult to effectively implement a complex environmental selection strategy. Therefore, in this paper, we improve the convergence and diversity of the population from the perspective of generation, which will reduce the computational burden of environmental selection strategy. Second, most methods manage both convergence and diversity of population together and use a single search strategy to guide population evolution, which causes these methods to have difficulty in maintaining convergence and diversity of population when faced with the huge search space. And they tend to fall into local optimality. Therefore, we propose a dynamic multi-population strategy with multiple search strategies in parallel to improve the search efficiency in a huge search space without increasing the population size to better maintain the diversity of the population.

### Large-scale optimization algorithms

Large-scale optimization algorithms can be divided into two categories based on their core ideas: decomposition-based and large-scale exploration-based.

Decomposition-based methods are based on the divide-and-conquer strategy, where the original problem is decomposed into smaller subproblems, and then each subproblem is optimized. Liu et al. [40] proposed a two alternative grouping strategy to divide high-dimensional decision variables. This strategy includes a convergence-related grouping strategy and a diversity-related grouping strategy, which will continuously alternate during population evolution, thus balancing the convergence and diversity of the population. A Bayesian-based parameter adjustment strategy is proposed to balance the accuracy and computational cost of the grouping strategy. However, all subproblems in the grouping strategy share the same decision space, which leads to the possibility that the subproblems do not search in all subspaces but converge on the same subspace, thus reducing the diversity of the solutions. Therefore, Yin and Cao [41] decomposed the decision space into many independent subspaces, each of which has at least one Pareto optimal solution, and individuals can only cross and mutate with other individuals in the same subspace, thus ensuring the diversity of the entire population.

The main idea of the large-scale exploration-based methods is to fully explore the high-dimensional decision space through many search strategies, thus optimizing all decision variables simultaneously. Qi et al. [42] proposed a two-stage multi-strategy search method. In the first stage, the population is divided into different levels by the objective function values of each individual. Different levels are updated by different strategies. Individuals with better objective function values focus on local exploitation, and individuals with poor objective function values focus on global exploration, thus ensuring that the population does not fall into local optimal. When entering the later stages of the search, the algorithm enters the second stage, the detailed search stage, where individuals at the same level learn from each other and search only locally to ensure population convergence. Wang et al. [43] proposed a superiority combination learning strategy based on the master-slave multi-subpopulation distributed model. The population is divided into subpopulations according to the objective function value, and the dominant subpopulation generates a learning particle for the inferior subpopulation to learn and evolve, which ensures sufficient communication and information exchange between different subpopulations and improves the diversity of the population. Gu et al. [44] proposed a chaotic differential strategy and a symmetric direction sampling strategy, which alternate during the evolution process to ensure convergence and diversity of the population. The symmetric direction sampling strategy increases the diversity of the search direction of the algorithm in the high-dimensional decision space by generating symmetric vectors of direction vectors to fully explore the high-dimensional decision space.

Decomposition-based methods require grouping of decision variables before starting the optimization. Therefore, the effectiveness of the grouping strategy directly affects the optimization results. Also, as the number of decision variables increases, the correlation between the decision variables becomes more complex, which requires more computational resources to group the decision variables, thus leading to a decrease in the computational resources allocated to the evolution. In contrast, the large-scale exploration-based methods fully explore the large-scale decision space to optimize all decision variables simultaneously through a multi-directional search strategy. At the same time, its computational cost does not increase significantly with the increase of decision variables. Therefore, when the decision variables are particularly large and the correlation is very complex, the large-scale exploration-based methods are more effective than the decomposition-based methods.

### Population-intelligent optimization algorithms

Many population-intelligent optimization algorithms (PIOAs) have been proposed, and many studies have shown that these algorithms outperform GA, DE in solving single-objective optimization problems [22–24]. There are also studies that extend the PIOAs to MOPs, which also outperform some traditional MOEAs [45,46]. But there exist two difficulties in the many-objectives extension of PIOAs: First, the search mechanism of the PIOAs is to select an individual with the optimal objective value to guide the movement of other individuals to realize the rapid convergence of the population to the current optimal position. Then the population starts to search the global optimal position from the current optimal position, which greatly improves the search efficiency of the population. In single-objective problems, the optimal individual is easy to choose. However, in MOPs, the decision of the optimal non-dominant individual requires additional computation due to the existence of dominance relationship, which results in MOEAs based on PIOAs to perform one more environmental selection than other MOEAs. Most of the current studies on multi-objective extension of PIOAs select the optimal nondominant individual by crowding degree [46], which results in that these methods have high computational complexity under the many-objective space and are far less efficient than other MaOEAs.Second, the convergence and diversity of the population need to be considered in MOPs, where convergence ensures that the population is close to the Pareto front and diversity ensures that the population can express the Pareto front as completely as possible. However, the search mechanism of the PIOAs leads to strong convergence and weak diversity of the population, and the gap between the two performances is more obvious in the many-objective space, which leads to the poor overall performance of MaOEAs based on PIOAs in MaOPs. Liang et al. [45] proposed a two-stage strategy to improve the sparrow search algorithm with many-objective and achieved good results. However, the study did not consider both large-scale and many-objective, while the algorithm performed poorly in terms of diversity.Therefore, this paper proposes a novel many-objective extension method of PIOAs, which uses the advantage of high convergence of PIOAs to manage the convergence of the population individually to avoid the disadvantage of poor diversity of PIOAs. And since the part of the method does not need to consider diversity, there is no need to select the individual with the best diversity performance as the leading individual, which effectively reduces the computational complexity of the PIOAs in the many-objective space.

The sparrow search algorithm (SSA) is a PIOA that simulates the predatory and anti-predatory behaviors of sparrow population [22]. Compared with other PIOAs, the SSA has two advantages: (1) stronger convergence; (2) the search strategy is a multi-directional search strategy, which is more suitable for dealing with large-scale optimization problems. In the SSA, the population is divided into three subpopulations: discoverers, followers, and vigilantes. The entire population is sorted according to the objective value. A certain percentage of individuals with superior objective values are assigned to the discoverer subpopulation and the rest of the individuals being assigned to the follower subpopulation. Vigilante subpopulation is randomly selected in the entire population. Both discoverers and followers can become vigilantes. Each subpopulation has its own search strategy.

The population is shown in :


$$\begin{array}{c} X=\left[\begin{array}{cccc} x_{11} & x_{12} & \cdots \; & x_{1d}\\ x_{21} & x_{22} & \cdots \; & x_{2d}\\ \vdots & \vdots & \ddots & \vdots \\ x_{n1} & x_{n2} & \cdots \; & x_{nd} \end{array}\right] \end{array}$$ (1)

*where* *n* is the population size and *d* is the dimension of decision variable.

The objective function is shown in :


$$\begin{array}{c} F_{X}=\left[\begin{array}{c} f([x_{11},x_{12},\ldots ,x_{1d}])\\ f([x_{21},x_{22},\ldots ,x_{2d}])\\ \vdots \\ f([x_{n1},x_{n2},\ldots ,x_{nd}]) \end{array}\right] \end{array}$$ (2)

The search strategy of discoverer subpopulation is shown in :


$$\begin{array}{c} x_{i}^{t+1}=\{\begin{array}{cc} x_{i}^{t}\cdot \exp\left(-\frac{i}{\alpha \cdot {\text{Iter}}_{\max}}\right),\; & \text{if }R<ST\\ x_{i}^{t}\cdot Q,\; & \text{if }R\geq ST \end{array} \end{array}$$ (3)

*where* *i* is the position of the individual in the population after sorting, *t* is the current iteration number, *α* ∈ ( 0 , 1 ) is a random number, ${\text{Iter}}_{\max}$ is the maximum number of iterations, *Q* is a random number obeying a normal distribution, *R* ∈ ( 0 , 1 ) is the risk value, and *ST* ∈ ( 0 . 5 , 1 ) is the risk threshold. The discoverer subpopulation will choose different search strategies according to the degree of the environmental risk. When *R* < *ST* means the degree of the environmental risk is low, the discoverer subpopulation will adopt a local exploitation strategy to fully search the local space; when *R* ≥ *ST* means the degree of environmental risk is high, the discoverer subpopulation will adopt a global exploration strategy to find other safe areas on a large space. The discoverer subpopulation is reordered internally after the search is completed.

The search strategy of the follower subpopulation is shown in :


$$\begin{array}{c} x_{i}^{t+1}=\{\begin{array}{cc} Q\cdot \exp\left(\frac{x_{\text{worst}}^{t}-x_{i}^{t}}{i^{2}}\right),\; & \text{if }i>n∕2\\ x_{\text{best}}^{t+1}+\left|x_{i}^{t}-x_{\text{best}}^{t+1}\right|\cdot A^{+},\; & \text{otherwise} \end{array} \end{array}$$ (4)

*where* $x_{\text{worst}}^{t}$ is the individual with the worst current objective value, $x_{\text{best}}^{t+1}$ is the individual with the optimal objective value after reordering within the discoverer subpopulation. $A^{+}=A^{T}{\left(AA^{T}\right)}^{-1}$, and *A* is a vector of 1 × *d* randomized to 1 or -1 in each dimension. When individuals are at the back of the population, they can only move as far away as possible from individuals in the worst position because they are farther away from the discoverer, whereas when individuals are at the front or middle of the population, they move towards the optimal position to get more food.

The search strategy of vigilante subpopulation is shown in :


$$\begin{array}{c} x_{i}^{t+1}=\{\begin{array}{cc} x_{\text{best}}^{t+1}+\beta \cdot \left|x_{i}^{t}-x_{\text{best}}^{t+1}\right|,\; & \text{if }f_{i}>f_{\text{best}}\\ x_{i}^{t}+K\cdot \left(\frac{\left|x_{i}^{t}-x_{\text{worst}}^{t}\right|}{\left(f_{i}-f_{\text{worst}}\right)+\epsilon }\right),\; & \text{if }f_{i}=f_{\text{best}} \end{array} \end{array}$$ (5)

*where* *β* is a random number obeying a normal distribution, $f_{i}$ is the value of the current individual’s objective function value, $f_{\text{best}}$ is the value of the optimal individual’s objective function value, $f_{\text{worst}}$ is the value of the worst individual’s objective value, *K* ∈ [ − 1 , 1 ] is a random number, and *K* ∈ [ − 1 , 1 ] is a constant that avoids the denominator being 0. The difference in vigilante subpopulation’s search strategies is mainly reflected in whether the optimal individual is a vigilante or not. When the optimal individual is *not* a vigilante, it represents that the optimal position of the population is safe, and the whole population will cluster towards the optimal position. But when the individual at the optimal position issues a vigilante, it means that the current optimal position is no longer safe, and the population will conduct a large-scale search to find a new optimal position, thus jumping out of the local optimal situation.

### Motivation

Although a large number of researches have proposed many excellent many-objective optimization algorithms and large-scale multi-objective optimization algorithms and achieved good results on the corresponding practical problems, the field of large-scale many-objective optimization algorithms is still in the development stage. Because large-scale many-objective optimization algorithms need to deal with high-dimensional decision variables and high-dimensional objectives at the same time, the performance requirements of the algorithms are higher, and thus the algorithm design will be more complex. For some *LSMaOEAs*, on the one hand, most of them still use the GA operator as the evolutionary operator, which leads to poor quality of the generated solutions and requires complex selection strategies to increase the selection pressure. And the *PIOAs* has been proven to be superior to the GA in the field of single-objective optimization and multi-objective optimization by several studies. However, in the field of many-objective optimization, there are a few studies on replacing GA with *PIOAs*. On the other hand, the decision variable grouping strategy consumes a large number of computational resources, which makes it difficult to balance the efficiency and accuracy. Especially in *LSMaOPs*, since high-dimensional objectives also need to be handled simultaneously, it is difficult to ensure the overall optimization efficiency of the algorithm by handling high-dimensional decision variables through a decomposition-based methods.

Based on the above motivation, this paper proposes a two-stage method based on SSA. The first stage accelerates the population convergence through many-objective SSA. The second stage fully explores the large-scale decision space through dynamic multi-population search strategy, so as to improve the diversity of the population as well as jumping out of the local optimal while ensuring the efficiency. The two stages alternate *adaptively* according to the population characteristics, thus balancing the convergence and diversity of the population.

## The proposed TS-SSA method

In this paper, we propose a two-stage method based on the sparrow search algorithm (SSA) and multi-population search strategy to solve large-scale many-objective optimization problems (*LSMaOPs*), called the two-stage sparrow search algorithm (*TS-SSA*). An *LSMaOP* can be defined as follows:

In *LSMaOPs*, maintaining a balance between convergence and diversity in such a high-dimensional decision space is crucial. To address this, our proposed method utilizes nondominated sorting, which ranks solutions based on Pareto dominance. Given *n*-dimensional decision variables $x=[x_{1},x_{2},\ldots ,x_{n}]\in {\mathbb{R}}^{n}$, the goal is to optimize *m* objective functions: $\text{Minimize/Maximize }F(x)=[f_{1}(x),f_{2}(x),\ldots ,f_{m}(x)]$, *where* *m* ≥ 4 represents the many objectives that need to be optimized simultaneously. A solution $x_{1}$ is said to dominate another solution $x_{2}$ if $x_{1}$ is no worse in all objectives and strictly better in at least one objective. The set of all nondominated solutions constitutes the Pareto front, representing the trade-offs among objectives where no objective can be improved without sacrificing another.

**Fig 1:**
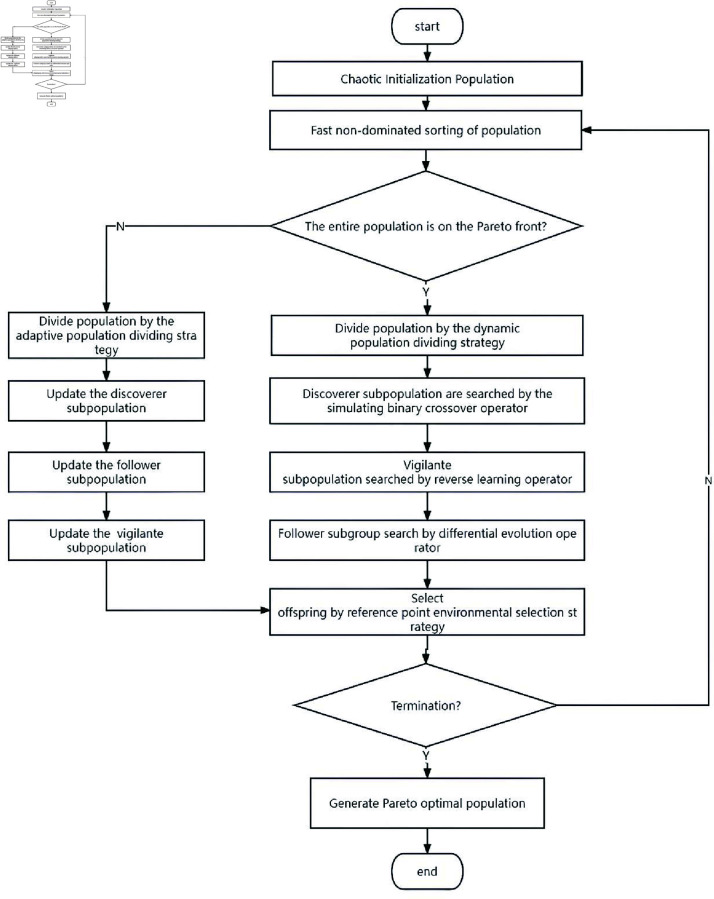
Procedure of TS-SSA.

We employ this formulation to drive the design of the *TS-SSA* algorithm, and the general flow of the method is shown in Fig 1. First, the population is chaotically initialized to ensure that the initial population is uniformly distributed in the space to improve the search efficiency of the method in the early stage. Then, the population is nondominated sorted, and if there are still individuals that have not converged to the Pareto front, the individuals on the Pareto front are classified into the discoverer subpopulation, the rest of the individuals in the population are classified into the follower subpopulation, and then some of the individuals in the whole population are randomly selected to be classified into the vigilant subpopulation. Each subpopulation is positionally updated by the improved search formula. When the whole population converges to the Pareto front, the size of the three subpopulations is divided according to the relationship between the vigilance value and the vigilance threshold. Each subpopulation is searched by the within-subpopulation search strategy. At the end of each search round, the optimal subpopulation is selected by the reference point environmental selection strategy.

The first stage mainly manages the convergence and ensures the fast convergence of the population to the PF. To enable SSA to be applied to many-objective optimization problems, this paper proposes an adaptive population dividing strategy and a random bootstrap search strategy. The adaptive population dividing strategy will divide the individuals in the optimal layer into the discoverer subpopulation and the rest into the follower subpopulation based on the results of the non-dominated ordering of the population. The random bootstrap search strategy enables the follower to randomly select a discoverer as the following target to search, thus achieving fast convergence of the population to the Pareto front. Meanwhile, in order to ensure the search efficiency of the algorithm in the early search stage, this paper proposes a chaotic population initialization strategy, so that the initial solutions of the population are guaranteed to be widely and randomly distributed as much as possible, thus reducing the ineffective search in the early search stage.

The second stage mainly manages the diversity of the population to ensure that the population is widely distributed on the PF. To fully explore the large-scale decision space, this paper proposes a dynamic multi-population search strategy. The original population is first divided into three smaller subpopulations by a dynamic population dividing strategy, and each subpopulation will adopt a different search strategy to accomplish a full exploration of the large-scale decision space. Among them, the discoverer subpopulation adopts the simulated binary cross search strategy to fully exploit the local space; the vigilant subpopulation adopts the reverse learning search strategy to detect the potential global optimal position to avoid the algorithm falling into the local optimal. The follower subpopulation adopts a differential search strategy and learns from the other two subpopulations through the learning factor to perform a comprehensive search.

The two stages will alternate adaptively according to the population characteristics so as to better balance the convergence and diversity of the population.

### Many-objective sparrow search algorithm

#### Population chaotic initialization.

Initializing the population by obeying uniformly distributed random numbers leads to insufficiently wide distribution of the population in the early stage, which reduces the search efficiency of the population in the early stage. Especially under the huge search space of *LSMaOPs*, a population that is not widely distributed tends to perform a large number of blind searches in the early stage, which affects the convergence of the population. Chaos theory describes the complex behavior of a nonlinear deterministic system with stochastic and ergodic properties [47]. The population generated by chaotic mapping can have a more uniform distribution in the space [48]. It has been proven on single-objective optimization problems that initializing the population by chaotic sequences can effectively improve the search efficiency of the algorithm in the early stage [49]. Therefore, in this paper, Singer mapping is chosen to generate chaotic sequences to initialize the population to improve the early search capability of the algorithm in the large-scale decision space. The Singer mapping is shown in :


$$\begin{array}{c} z_{k+1}=\mu (7.86z_{k}-23.312z_{k}^{2}+28.752z_{k}^{3}-13.302875z_{k}^{4}) \end{array}$$ (6)

*where* the Singer mapping has chaotic properties for *μ* ∈ [ 0 . 9 , 1 . 08 ] . Singer mappings have a larger value domain than other chaotic mappings, which can accommodate more types of problems. In this paper, *μ* is set to 1.

#### Adaptive population dividing strategy.

The size of the subpopulation in the original SSA is generally subjective, with 20% to 40% of the population being discoverers and the rest being followers. Vigilantes make up 10 to 20 percent of the population. However, in *MaOPs*, a fixed dividing strategy may result in dividing nondominant individuals into the follower subpopulation, which causes convergent aggregation among nondominant individuals and reduces the diversity of the population, thus incurring additional computational cost, or dividing dominant individuals into the discoverer subpopulation, which causes dominant individuals to fail to converge toward the Pareto front, thus reducing the convergence efficiency. Both of these situations reduce the optimization efficiency of the algorithm. Therefore, this paper proposes an adaptive population dividing strategy that first performs a fast non-dominated sorting of the population, and then all non-dominated individuals at the current Pareto front are divided into the discoverer subpopulation, and the remaining dominated individuals are divided into the follower subpopulation. Non-dominated sorting is a method used to evaluate and rank solutions, particularly within the context of multi-objective genetic algorithms (*MOGAs*). The technique involves categorizing solutions into different levels or fronts. The first front includes all non-dominated solutions—that is, those solutions that are not inferior to any other solution across all objectives. Subsequent fronts include solutions that are dominated only by the solutions in the preceding front. This hierarchical ranking facilitates the algorithm’s focus on solutions close to the Pareto front while maintaining diversity within the population. The Pareto front is a concept central to multi-objective optimization problems. It refers to the set of solutions where no other solution exists that improves one objective without worsening another. Formally, for a given set of solutions *SS* in a multi-objective optimization problem with objectives $f_{1},f_{2},\ldots ,f_{m}$, a solution *x* ∈ *S* is said to be on the Pareto front if there does not exist any other solution *y* ∈ *S* such that $f_{i}(y)\leq f_{i}(x)$ for all *i* and $f_{j}(y)<f_{j}(x)$ for at least one *j*. Solutions on the Pareto front are considered “optimal” because improving one objective function will necessarily result in a degradation of performance in at least one other objective.

The discoverer subpopulation will randomly wander on the Pareto front, which can increase the diversity of the population while avoiding falling into a local optimal. The follower subpopulation will converge rapidly toward the Pareto front by random bootstrap search strategy. It makes little sense for the follower subpopulation to engage in local exploitation or global exploration, since none of the follower subpopulation are at the Pareto front, so in most cases the explored positions may not be as good as the current Pareto front, which leads to a large number of pointless blind searches that add to the computational cost. Therefore, this paper lets the follower subpopulation quickly converge to the discoverer subpopulation, so that more individuals can search on the Pareto front, thus improving the search efficiency. In the original SSA, the vigilante subpopulation is randomly selected from the whole population, and both discoverers and followers have the chance to become vigilantes. However, in the multi-objective optimization problem, the follower only increases the cost of blind search because it is not on the current PF. Therefore, in the adaptive population partitioning strategy, the vigilance will not be selected from the followers, but only randomly from the finders. At the same time, because the alert is only selected from the finders, this paper also expands the selection ratio of alert, and adaptively adjusts the proportion of alert according to :


$$\begin{array}{c} rov=\alpha +\beta \cdot \tanh\left(\gamma +\sigma \cdot \frac{\text{iter}}{{\text{iter}}_{\max}}\right) \end{array}$$ (7)

*where*, the *rov* proportion is alert, iter is the current number of iterations, ${\text{iter}}_{\max}$ is the maximum number of iterations, *α*, *β*, *γ*, *σ* is super parameters.

#### Random bootstrap search strategy.

In the original SSA, the followers will converge to the individual with the optimal objective function value. Thus, most studies on multi-objective improvement of SSA will determine the optimal non-dominated individual by calculating the crowded distance [46,50]. Calculating crowded distances with high-dimensional objectives has a very high computational complexity, which also makes it impossible to extend SSA to many-objectives with this strategy. Therefore, this paper proposes a stochastic bootstrap search strategy that can guarantee fast convergence of the population in a many-objective space with low computational complexity.

The random bootstrap search strategy for the follower subpopulation is shown in :


$$\begin{array}{c} x_{i}^{t+1}=\{\begin{array}{cc} Q\cdot \exp\left(\frac{x_{\text{rf}}^{t}-x_{i}^{t}}{i^{2}}\right),\; & \text{if }i>\frac{n}{2}\text{ and }{\text{Subpop}}_{\text{discoverer}}<\frac{n}{2}\\ x_{\text{rd}}^{t+1}+\left|x_{i}^{t}-x_{\text{rd}}^{t+1}\right|\cdot A^{+}\cdot L,\; & \text{otherwise} \end{array} \end{array}$$ (8)

*where* $x_{\text{rf}}$ is randomly selected individual in the follower subpopulation other than individual *i*. $x_{\text{rd}}^{t+1}$ is randomly selected individual in the discoverer subpopulation. *L* is a 1 × *d* vector, $A^{+}=A^{T}{\left(AA^{T}\right)}^{-1}$, and *A* is a vector of 1 × *d* randomized to 1 or -1 in each dimension, *n* is the population size, ${\text{Subpop}}_{\text{discoverer}}$ is discoverer subpopulation size. SSA itself has excellent convergence, but the diversity of SSA performs poorly due to its search and bootstrapping mechanisms. In contrast, MOPs need to consider both convergence and diversity. Therefore, other studies on multi-objective improvement of SSA, since SSA is required to manage both convergence and diversity, these studies can only maximize the diversity performance of SSA by selecting the optimal nondominant individual through the crowded distance.

The disadvantage of this is that the optimal nondominant individual will also lead other nondominant individuals to itself, which destroys the broad distribution of the population on the Pareto front, which is not conducive to managing diversity, and the computation of crowded distances is more expensive in the high-dimensional objective space. However, in *TS-SSA*, convergence and diversity are managed separately, and SSA only needs to mainly manage convergence primarily to ensure that the population converges quickly to the Pareto front, and then increase the population diversity as much as possible. Therefore, randomly selecting any individuals on the Pareto front can ensure fast convergence of the dominant individuals. It also avoids the high computational cost of selecting the optimal nondominant individual by computing the crowded distance. When the followers are at the back of the population and the number of individuals in the discoverer subpopulation does not exceed half of the total number of the population, i.e., $i>\frac{n}{2}$ and ${\text{Subpop}}_{\text{discoverer}}<\frac{n}{2}$, it is better to stay away from other followers. Because there are a few discoverers at this time, guiding the followers at this time will lead to the aggregation of the population and reduce the diversity of the population.

The random bootstrap search strategy for the vigilante subpopulation is shown in :


$$\begin{array}{c} x_{i}^{t+1}=\{\begin{array}{cc} x_{\text{rd}}^{t}+\beta \cdot \left|x_{i}^{t}-x_{\text{rd}}^{t}\right|,\; & \text{if }\;{\text{Subpop}}_{\text{discoverer}}<\frac{n}{2}\\ x_{i}^{t}+K\cdot \left(\frac{\left|x_{i}^{t}-x_{\text{rf}}^{t}\right|}{\frac{1}{M}\overset{M}{\underset{j=1}{\sum }}(f_{i,j}-f_{\text{rf},j})+\epsilon }\right),\; & \text{if }\;{\text{Subpop}}_{\text{discoverer}}>\frac{n}{2} \end{array} \end{array}$$ (9)

*where* *β* is a random number obeying a normal distribution, *M* is the number of objectives, $f_{i}$ is the value of the current individual’s objective function value, $f_{\text{rf}}$ is the value of the random individual’s objective function value of follower subpopulation, *K* ∈ [ − 1 , 1 ] is a random number, and *K* ∈ [ − 1 , 1 ] is a constant that avoids the denominator being 0. Because all vigilantes are selected from the discoverer subpopulation, and if there are a few individuals in the discoverer subpopulation, the number of vigilantes will be small. Thus, the vigilantes will randomly interact with each other for local exploitation. When there are many individuals in the discoverer subpopulation, the number of vigilantes also increases, at which point the vigilantes randomly select followers to communicate with, thus completing the global exploration in search of a potential Pareto optimal front. The discoverer subpopulation is still searched according to .

### Dynamic multi-population search strategy

After the population has all converged to the Pareto front, limited by the performance of SSA, the diversity of the population is poor at this point, while it is difficult to increase the diversity of the population through SSA again. Therefore, this paper proposes a dynamic multi-population search strategy based on the SSA to mainly manage the diversity. The dynamic multi-population search strategy includes the dynamic population dividing strategy and the multi-population search strategy. First, the dynamic population dividing strategy divides the population into three subpopulations: discoverer subpopulation, follower subpopulation, and vigilante subpopulation. The size of each subpopulation is dynamically adjusted, which allows the main search direction of the population to change dynamically for full exploration. For the multi-population search strategy, each subpopulation performs a different search strategy in different directions, thus fully exploring the large-scale decision space, searching for the potential Pareto optimal front, and increasing the diversity of the population.

#### Dynamic population dividing strategy.

In the original SSA, the risk value *R* represents the population’s risk assessment of the environment, and the risk threshold *ST* represents the population’s risk tolerance. The dynamic change of *R* can constantly adjust the search direction of the population, thus avoiding the algorithm falling into a local optimal.

The dynamic population dividing strategy is shown in :


$$\begin{array}{c} \{\begin{array}{cc} {\text{SubPop}}_{\text{discoverer}}=n∕2\;\\ {\text{SubPop}}_{\text{follower}}=n∕4\;\\ {\text{SubPop}}_{\text{vigilante}}=n∕4\; & \text{if }R<ST\\ {\text{SubPop}}_{\text{discoverer}}=n∕4\;\\ {\text{SubPop}}_{\text{follower}}=n∕4\;\\ {\text{SubPop}}_{\text{vigilante}}=n∕2\; & \text{if }R\geq ST \end{array} \end{array}$$ (10)

*where* ${\text{SubPop}}_{\text{discoverer}}$ is the discoverer subpopulation size, ${\text{SubPop}}_{\text{follower}}$ is the follower subpopulation size, ${\text{SubPop}}_{\text{vigilante}}$ is the vigilante subpopulation size, *n* is the population size. The discoverer subpopulation performs a local exploitation, the vigilante subpopulation performs a global exploration, and the follower subpopulation learns the search information of the other two subpopulations. Therefore, when *R* < *ST*, i.e., when the population is currently in a more secure position, the discoverer subpopulation expands and the population performs a local exploitation to increase the diversity of the population at the local level. When *R* ≥ *ST*, i.e., the current position of the population is no longer safe, the vigilante subpopulation expands and the population mainly performs a global exploration for potentially optimal positions to avoid falling into a local optimal and to improve the global diversity of the population. Dynamic changes in subpopulation size change the dominant search direction of the population, leading to multi-directional search that allows full exploration of large-scale decision spaces.

However, *R* in the original SSA obeys a uniform distribution, which is difficult to satisfy the randomness of dynamic changes. Therefore, this paper also applies Singer mapping on *R* to generate a set of chaotic sequences to ensure the randomness and traversal of *R*, so as to ensure the randomness of the dynamic dividing of the population. Second, the *ST* in the original SSA is a fixed preset value, which does not fully take into account the different needs of the algorithm’s early and late search stages. In the early search stage, there is still a large amount of potential space unexplored, so global exploration should be the main focus at this time; while in the late optimization stage, most of the space has been explored, and local exploitation should be the main focus at this time.

Therefore, the *ST* in this paper is adaptively adjusted according to :


$$\begin{array}{c} ST=\frac{ST_{\text{max}}+2ST_{\text{min}}}{3}+\frac{ST_{\text{max}}-ST_{\text{min}}}{3}\cdot \tanh\left(-5+10\cdot \frac{\text{iter}}{{\text{iter}}_{\text{max}}}\right) \end{array}$$ (11)

**Fig 2:**
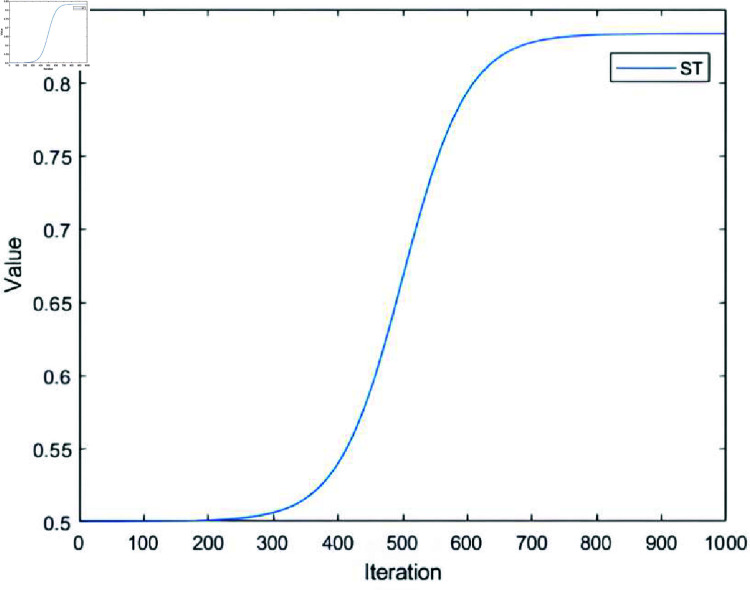
ST Change.

*where* $ST_{\text{max}}$ is the maximum value of *ST*, $ST_{\text{min}}$ is the minimum value of *ST*, iter is the current iteration number. Fig 2 shows the change of *ST* when $ST_{\text{max}}=1$, $ST_{\text{min}}=0.5$, ${\text{iter}}_{\text{max}}=1000$. In the early search stage, we keep *ST* at a low value to increase the frequency of dynamic dividing of the population, so that the main search direction of the population is constantly changing to full local exploit and global explore. As the search continues, *ST* will gradually increase to allow the discoverer subpopulation to gradually become dominant. In the later search stage, *ST* is kept at a high level so that the discoverer subpopulation becomes the dominant subpopulation of the population, which results in the entire population being dominated by local exploitation in the later search stage.

#### Multi-population search strategy.

In order to fully explore the large-scale decision space, avoid falling into the local optimal, and search for potential Pareto optimal front, this paper proposes a multi-population search strategy that dynamically changes the search direction during the optimization process to achieve full local exploitation and global exploration. The discoverer subpopulation adopts the simulated binary cross search strategy (SBX) for local exploitation. The vigilante subpopulation adopts the reverse learning search strategy (RL) for global exploration. The follower subpopulation adopts the differential search strategy (DE) for comprehensive exploration.

The SBX operator used by the discoverer subpopulation is a search strategy that simulates a single-point binary crossover. The parent $x^{1}$, $x^{2}$ are shown in :


$$\begin{array}{c} \{\begin{array}{c} x^{1}=\{x_{1}^{1},x_{2}^{1},\ldots ,x_{d}^{1}\}\;\\ x^{2}=\{x_{1}^{2},x_{2}^{2},\ldots ,x_{d}^{2}\}\; \end{array} \end{array}$$ (12)

The children $c^{1}$, $c^{2}$ are then generated by the crossover of :


$$\begin{array}{c} \{\begin{array}{c} c^{1}=0.5*\left[(1+\beta )*x^{1}+(1-\beta )*x^{2}\right]\;\\ c^{2}=0.5*\left[(1-\beta )*x^{1}+(1+\beta )*x^{2}\right]\; \end{array} \end{array}$$ (13)

*where* *β* is determined by the distribution factor *η* according to :


$$\begin{array}{c} \beta =\{\begin{array}{cc} {\left(\text{rand}*2\right)}^{1∕(1+\eta )},\; & \text{if }\text{rand}\leq 0.5\\ {\left(\frac{1}{\left(2-\text{rand}*2\right)}\right)}^{1∕(1+\eta )},\; & \text{otherwise} \end{array} \end{array}$$ (14)

*η* is a user-specified cross-distribution index that controls the degree of similarity between the parent and children; the larger *η* is, the more similar the children are to the parent, which causes SBX to favor local exploitation. The smaller *η* is, the less similar the children are to the parent, which causes SBX to favor global exploration. However, SBX is essentially an information exchange between individuals of a population, so the local exploitation ability is stronger. Therefore, in this paper, the cross-distribution index is set larger to fully local exploit.

The RL operator used by the vigilante subpopulation is based on :


$$\begin{array}{c} c=\text{upper}+\text{lower}-x \end{array}$$ (15)

*where* upper is the upper limit of the decision variables and lower is the lower limit of the decision variables. RL operator is computationally simple but changes rapidly and can be searched over a wide range. In single-objective optimization problems, RL operator is destructive to population convergence. Constraints are usually imposed to reduce the destructiveness of RL operator on population convergence. The most common constraint method is to decide whether to keep RL individuals or not by computing and comparing objective values. However, in *LSMaOPs*, the additional computation and comparison of objective values is more consuming, so in this paper we constrain them by *R*, *ST* and two other subpopulations. Compared with other search operators, the RL operator performs well on the diversity and can perform more extensive and faster search. Especially in *LSMaOPs*, RL operator can be more efficient to perform fast and extensive search under huge search spaces. Moreover, in this paper, RL only needs to manage diversity, which reduces its impact on convergence.

DE operator used by the follower subpopulation is a search strategy based on differential information between individuals. It has strong search ability in complex environments. DE operator in this paper is realized based on :


$$\begin{array}{c} c_{\text{DE}}=x_{\text{DE}}+\alpha *\left(c_{\text{SBX}}^{1}-c_{\text{SBX}}^{2}\right)+(1-\alpha )*\left(c_{\text{RL}}^{1}-c_{\text{RL}}^{2}\right) \end{array}$$ (16)

*where* $c_{\text{SBX}}^{1}$, $c_{\text{SBX}}^{2}$ are two random individuals in the offspring produced by the discoverer subpopulation, and $c_{\text{RL}}^{1}$, $c_{\text{RL}}^{2}$ are two random individuals in the offspring produced by the vigilante subpopulation. *α* is the learning factor, which controls how much the follower subpopulation learns about the other two subpopulations, and is defined in :


$$\begin{array}{c} \alpha =0.5+\left(1-\frac{R}{ST}\right)*\left(\exp\left(1-\frac{\text{iter}}{{\text{iter}}_{\max}}\right)-1\right) \end{array}$$ (17)

*α* is automatically adjusted according to the environmental risk and the number of iterations. When *R* > *ST*, i.e., the environmental risk is high, the follower subpopulation is more inclined to learn the vigilante subpopulation and adopt global exploration. On the contrary, when *R* ≤ *ST*, i.e., the environmental risk is low, the follower subpopulation is more inclined to learn the discoverer subpopulation and adopt conservative search. At the same time, as the search process into the later stage, the follower subpopulation chooses a more balanced learning rate for a comprehensive search. Also, considering that the discoverer subpopulation and the vigilante subpopulation are unequal in size, to ensure traversal ability, the already selected individuals are removed from the set, and when the set is empty, all the removed individuals of that subpopulation are added to the set again. The follower subpopulation performs its own search by learning the differential information of the offspring of the other two subpopulations, while continuously adjusting its search strategy to improve the diversity of the subpopulation.

### Environmental selection strategy

In this paper, we use a reference point environmental selection strategy similar to *NSGA-III* [12]. In many-objective space, this method has lower computational complexity and stronger selection pressure compared to the environment selection strategy by the degree of crowding.

First, we perform a fast non-dominated sorting of the mixed population of parents and children into different Pareto layers $F_{i}(i=1,2,\ldots ,n)$. If $|F_{1}|=n$, the individuals in $F_{1}$ are placed in $S_{\text{next}}$ and go directly to the next iteration. If $|F_{1}|>n$, the reference point selection strategy is applied in $F_{1}$ and *NP* individuals are selected to be put into $S_{\text{next}}$. If $|F_{1}|<n$, the individuals in each layer are put into $S_{\text{next}}$ in order, until ${⋃}_{i=1}^{l}F_{i}\geq NP$. And then $|F_{l}|-{⋃}_{i=1}^{l}F_{i}+n$ individuals are selected from $F_{l}$ into $S_{\text{next}}$.

In this paper, compared with the complex environmental selection strategy in other studies, we do not make much improvement in the environmental selection strategy. We choose a simpler strategy with low computational complexity. Since *TS-SSA* produces population with good convergence and diversity, it is not necessary to give too much selection pressure in the environmental selection strategy to select the better offspring to ensure the overall performance of the population.

The pseudo-code for *TS-SSA* is shown in Algorithm 1.

**Algorithm 1. TS-SSA.**

**Input:** *N*: population size, *M*: number of objective functions, *D*: number of decision variables, *l*ower: lower limit of the decision variable, *u*pper: upper limit of the decision variable.

**Output:** Optimal solutions *Pop*.

1: *Pop ← chaosInitialization(N, M, D)*

2: *R ← rand()*

3: **while** termination criterion not fulfilled **do**

4:  *R ← chaosMapping*(*R*)

5:  *ST ← adaptionST*()

6:  {*Subpop_discoverer_, Subpop_follower_*} ← *NDSort* (*Pop*)

7:  **if** *Subpop_discoverer_* ≠ *N* **then**

8:   *Offspring_discoverer_ ← MaOSSAdiscoverer*(*Subpopdiscoverer*)

9:   *Offspring_follower_* ← *MaOSSA_follower_*(*Subpop_follower_*)

10:   *Subpop_vigilante_* ← *randomSelection*(*Subpop_discoverer_*/5)

11:   *Offspring_vigilante_* ← *MaOSSA_vigilante_*(*Subpop_vigilante_)*

12:  **else**

13:   **if** *R < ST* **then**

14:    *Subpop_discoverer_* ← *randomSelection*(*Pop, N*/2)

15:    *Subpop_follower_* ← *randomSelection*(*Pop \ Subpop_discoverer_, N*/4)

16:    *Subpop_vigilante_* ← *Pop* \ (*Subpop_discoverer_* ∪ *Subpop_follower_*)

17:   **else**

18:    *Subpop_vigilante_* ← *random_Selection_*(*Pop, N*/2)

19:    *Subpop_follower_* ← *random_Selection_*(*Pop \ Subpop_vigilante_, N*/4)

20:    *Subpop_discoverer_* ← *Pop* \ (*Subpop_vigilante_* ∪ *Subpop_follower_*)

21:   **end if**

22:  **end if**

23:  *Offspring_discoverer_* ← *SBX*(*Subpop_discoverer_*)

24:  *Offspring_vigilante_* ← *RL*(*Subpop_vigilante_*)

25:  *Offspring_follower_* ← *DE*(*Subpop_follower_*)

26:  *Offspring ← Offspring_discoverer_* ∪ *Offspring_follower_* ∪ *Offspring_vigilante_*

27:  *Pop ← EnvironmentalSelection*(*Pop, Offspring*)

28: **end while**

In summary, the overall best time complexity of *TS-SSA* is *O* ( *MNlog* ⁡ *N* ) and the worst time complexity is $O(MN^{2})$.

## Experimental results and discussions

In this section, we will verify the effectiveness of the proposed method through a series of experiments. Benchmark problems, performance metric, parameter settings, and experimental results and analysis are described next.

### Experimental design

#### Benchmark problems.

The benchmark problems used in this paper include DTLZ1-7 [52], IDTLZ1-2 [53], SDLTZ1-2 [12] and LSMOP1-9 [54] to evaluate the performance and effectiveness of the proposed method. These problems have different Pareto fronts and characteristics, which can better evaluate the overall performance of the method on different types of problems. Table 1 shows the settings of the relevant parameters of the benchmark problems.

**Table 1:** Parameters related to benchmark problems.

	DTLZ1-7	IDLZ1-2	SDTLZ1-2	LSMOP1-9
M	3/8/10/15/20	3/8/10/15/20	3/8/10/15/20	3/8/10/15/20
D	300/800/1000/	300/800/1000/	300/800/1000/	300/800/1000/
	1500/2000	1500/2000	1500/2000	1500/2000

#### Performance metric.

The inverse iteration distance (IGD) is chosen to evaluate the overall performance of the method [55]. The IGD is calculated as shown in :


$$\begin{array}{c} \text{IGD}(PF)=\frac{1}{\left|PF^{*}\right|}\underset{z^{*}\in PF^{*}}{\sum }\text{minDistance}(z^{*},PF) \end{array}$$ (18)

*where* *PF* is the approximate Pareto optimal front computed by the method, $PF^{*}$ is the true Pareto front, $z^{*}$ is the individual in $PF^{*}$, and minDistance(z*, *PF*) computes the minimum Euclidean distance from individual $z^{*}$ to *PF*. IGD evaluates the overall performance of the methods by calculating the average of the minimum distances from the set of points on the true Pareto front to the approximate Pareto optimal front. Therefore, IGD is effective in comprehensively evaluating the convergence and diversity of the solution set, if the true Pareto front is known.

#### Parameter settings.

To verify the effectiveness of the method proposed in this paper, ten advanced MOEAs, FDV [56], LMOEADS [57], IMMOEAD [58], DGEA [59], LERD [60], MOCGDE [61], AGEMOEAII [62], HEA [63], SGECF [64], and UCLMO [65], are selected for comparison. The experiments are performed on a computer with hardware configuration of Intel Core I7-11800 @2.30 GHz and 32 GB RAM. The program was written in MATLAB R2023a. All experiments are performed on the open source MOEA platform PlatEMO 4.2 [66], where some of the relevant code is available (This data can be achieved by visiting https://github.com/BIMK/PlatEMO).

TS-SSA (This data can be achieved by visiting https://figshare.com/s/2cb32f7acb4b6cc3e27e) uses the environmental selection strategy based on reference points, so the selection of reference points in the experiment was obtained through the internal and external double layer sampling method. The population size is set as shown in Table 2. All methods will use the same population size for fair comparisons. The hyperparameter in is chosen as *α* = 0 . 3, *β* = − 0 . 2, *γ* = 10, *σ* = 10.

**Table 2:** Population size.

*M*	Population size	MaxFEs
3	92	92000
8	156	156000
10	276	276000
15	420	420000
20	652	652000

As shown in Table 2, the maximum number of the function evaluations (*MaxFEs*) is the population size multiplied by 1000. For example, the population size of the 8-objective problem is 156, so the maximum number of the evaluation function is 156000. To ensure fairness, all algorithms will have the same maximum number of function evaluations. Each method is run 30 times on each problem, and the mean and standard deviation of the performance metric are taken for statistical analysis and comparison. The statistical metric used for the experiments is the Mann-Whitney-Wilcoxon rank sum test [67] with a 5% significance level.

Most of the methods used simulated binary crossover operators and polynomial variational operators, so for a fair comparison, the distribution indices of all crossover and variational operators are set to 20. For some methods that require special parameters to be set individually, the experiments are set to the optimal reference values given in the original paper.

### Experimental results and analysis

#### Results and analysis of comparisons with other methods.

First, we conducted comparative experiments between *TS-SSA* and 10 other state-of-the-art methods on 5 dimensions and 20 benchmark problems, and the results of the IGD metric are shown in Tables 3, 4, 5, 6, and 7. In each table, the first four columns are the benchmark problems, the population size, the number of objectives, and the dimensions of the decision variables. The mean of the metric results for the 30 runs is shown outside the parentheses, and the standard deviation is shown in parentheses. Bold numbers indicate the optimal results of the benchmark problems. The symbols “+”, “-” and “=” indicate whether the null hypothesis of the results, which are generated by *TS-SSA* and compared methods, is accepted or rejected with the significance level 5% by the Mann-Whitney-Wilcoxon rank sum test. The last row of each table gives the summed results of the rank sum test, where the three numbers from left to right indicate the number of times the method is better, worse, and equal compared to the method proposed in this paper.

**Table 3:** IGD metric of different methods on 3-objective benchmark problems.

Problem	N	M	D	FDV	LMOEADS	IMMOEAD	DGEA	LERD	MOCGDE	AGEMOEAII	HEA	SGECF	UCLMO	TS-SSA
DTLZ1	92	3	300	1.7458e+1 (3.39e+0) -	1.2250e+0 (1.95e+0) =	4.0840e+3 (2.69e+2) -	9.1873e+2 (4.37e+2) -	4.5321e+2 (2.88e+2) -	1.6460e+2 (2.10e+2) -	6.6935e+2 (3.20e+1) -	7.1188e+2 (3.75e+1) -	2.0734e+3 (1.95e+1) -	4.5437e+3 (2.00e+2) -	**1.1322e+0** (**8.29e+0**)
DTLZ2	92	3	300	3.1365e+1 (6.14e-2) -	2.3948e-1 (2.19e-2) -	3.0252e+0 (2.03e-1) -	2.4790e+0 (3.32e-1) -	8.5804e-1 (1.63e-1) -	5.6893e-2 (5.54e-4) =	6.6731e-2 (2.02e-3) -	6.9930e-2 (2.06e-3) -	1.0071e+0 (8.67e-2) -	6.3594e-2 (1.22e-3) -	**5.5160e-2** (**5.68e-4**)
DTLZ3	92	3	300	5.2981e+1 (1.34e+1) -	**6.2044e+0** (**1.36e+1**) +	9.8585e+3 (3.32e+2) -	2.1201e+3 (1.62e+3) -	8.0707e+2 (9.02e+2) -	2.1082e+2 (3.25e+2) -	2.2424e+3 (1.22e+2) -	2.1299e+3 (1.19e+2) -	7.0335e+3 (7.16e+1) -	1.5231e+4 (4.30e+2) -	2.7518e+1 (1.25e+2)
DTLZ4	92	3	300	2.9351e+1 (2.26e-1) -	2.9876e-1 (1.63e-1) -	5.0951e+0 (7.44e-1) -	3.2680e+0 (9.47e-1) -	4.2581e-1 (3.54e-1) -	4.4986e-1 (7.66e-2) +	1.9060e-1 (2.53e-1) -	4.0514e-1 (2.25e-1) +	1.2335e+0 (1.02e+0) -	1.1632e-1 (1.50e-1) +	5.4707e-1 (3.26e-4)
DTLZ5	92	3	300	1.6288e+1 (3.93e-2) -	2.3936e-1 (2.57e-2) -	3.3161e+0 (6.10e-1) -	2.3481e+0 (3.61e-1) -	6.7998e-1 (1.76e-1) -	**4.5818e-3** (**3.61e-5**) +	2.4428e-2 (2.12e-3) -	4.0303e-2 (2.79e-3) -	1.2719e+0 (2.13e-1) -	8.9309e-2 (3.86e-3) -	1.3376e-2 (1.91e-3)
DTLZ6	92	3	300	8.4894e+1 (1.70e+1) -	6.4501e-3 (5.97e-4)+	2.2805e+2 (2.97e+0) -	1.0344e+2 (2.52e+1) -	2.3858e-2 (5.30e-4) -	**4.5395e-3** (**4.85e-5**) +	1.1062e+2 (4.17e+0) -	1.3379e+2 (3.91e+0) -	4.5534e+3 (6.21e-5) +	2.4606e+2 (1.80e+0) -	1.7445e-2 (2.55e-3)
DTLZ7	92	3	300	2.5718e+0 (1.36e+0) -	8.2131e-2 (4.59e-3)+	3.7979e+0 (7.15e-1) -	7.2392e+0 (1.32e+0) -	9.7416e-1 (2.77e-1)+	6.0457e-1 (6.92e-1) +	1.2647e-1 (2.26e-2) +	1.1224e-1 (1.17e-2) +	1.0871e+0 (1.02e-1) -	2.4739e-1 (1.62e-2) +	1.1189e-1 (1.52e-2)
IDTLZ1	92	3	300	2.6758e+1 (6.93e+0) -	3.4090e+0 (4.98e+0) -	6.3757e+3 (3.70e+2) -	1.3232e+3 (6.72e+2) -	3.6886e+2 (4.63e+2) -	1.1292e+3 (9.56e+2) -	1.3237e+3 (7.28e+1) -	1.2657e+3 (7.96e+1) -	4.0668e+3 (4.83e+1) -	1.4743e+3 (1.48e+2) -	**1.1669e-1** (**9.56e-2**)
IDTLZ2	92	3	300	1.5884e+1 (1.68e-2) -	2.4643e-1 (1.76e-2) -	2.5659e+0 (2.51e-1) -	1.6703e+0 (5.26e-1) -	8.7498e-1 (2.20e-1) -	5.6670e-2 (5.15e-4) -	7.6559e-2 (1.85e-3) -	8.8791e-2 (2.44e-3) -	1.0672e+0 (1.42e-1) -	7.0748e-2 (5.99e-4) -	**3.6758e-2** (**3.06e-3**)
SDTLZ1	92	3	300	1.9363e+1 (5.22e+0) -	**2.0717e+0** (**3.21e+0**) =	5.1441e+3 (3.87e+2) -	1.4752e+3 (6.52e+2) -	7.1242e+2 (6.86e+2) -	3.7514e+2 (6.67e+2) -	1.0424e+3 (4.94e+1) -	1.0894e+3 (6.22e+1) -	3.1474e+3 (3.01e+1) -	6.7500e+3 (2.21e+2) -	2.5475e+0 (1.67e+1)
SDTLZ2	92	3	300	4.8705e-1 (8.01e-2) -	**4.4452e-1** (**4.66e-2**) -	3.5268e+0 (1.88e-1) -	3.0280e+0 (4.26e-1) -	1.3515e+0 (3.53e-1) -	1.2772e-1 (1.44e-3) -	1.5233e-1 (4.29e-3) -	1.6020e-1 (4.02e-3) -	1.3019e+0 (1.45e-1) -	1.6415e-1 (1.49e-3) -	1.1217e-1 (1.29e-3)
LSMOP1	92	3	300	2.8960e-1 (3.47e-2) -	5.2693e-1 (2.80e-2) -	3.1342e+0 (6.42e-1) -	7.1189e-1 (2.31e-1) -	1.2705e+0 (5.24e-1) -	**1.1657e-1** (**4.39e-2**) =	6.9113e-1 (1.44e-1) -	3.9711e-1 (5.94e-2) -	8.6072e-1 (0.00e+0) -	3.6849e-1 (3.02e-2) -	1.5135e-1 (1.49e-1)
LSMOP2	92	3	300	5.9836e-2 (3.24e-3) -	6.0789e-2 (1.88e-3) -	9.9794e-2 (9.38e-4) -	7.2833e-2 (1.01e-2) -	9.2840e-2 (2.13e-3) -	8.1422e-2 (4.14e-3) -	7.9615e-2 (9.99e-4) -	8.1609e-2 (9.13e-4) -	9.5815e-2 (8.08e-4) -	8.1254e-2 (4.64e-4) -	**5.8413e-2** (**4.74e-3**)
LSMOP3	92	3	300	9.1881e-1 (2.40e-1) -	**8.5350e-1** (**2.05e-2**) =	7.6228e+0 (1.54e+0) -	9.5804e-1 (6.43e-1) =	9.5663e+0 (1.12e+0) -	1.4385e+1 (3.21e+1) -	3.2711e+0 (6.60e-1) -	3.1631e+0 (1.51e+0) -	8.6072e-1 (0.00e+0) =	2.0988e+0 (1.47e-1) -	8.5897e-1 (8.88e-3)
LSMOP4	92	3	300	1.4773e-1 (1.07e-2) -	1.6499e-1 (5.01e-3) -	2.7666e-1 (3.98e-3) -	2.0023e-1 (3.59e-2) -	2.2714e-1 (1.61e-2) -	2.5418e-1 (1.69e-2) -	1.9002e-1 (3.74e-3) -	1.8847e-1 (4.46e-3) -	2.5524e-1 (7.35e-3) -	2.4840e-1 (3.81e-3) -	**1.3595e-1** (**1.63e-2**)
LSMOP5	92	3	300	5.4042e-1 (3.11e-1) -	5.1162e-1 (2.35e-2) -	1.6342e+0 (2.50e-1) -	7.7576e-1 (5.24e-1) -	1.8347e+0 (1.41e+0) -	**3.3493e-1** (**1.31e-1**) +	9.9046e-1 (2.69e-1) -	7.2527e-1 (1.19e-1) -	7.0287e-1 (2.09e-1) =	6.0243e-1 (4.31e-2) -	3.3657e-1 (1.10e-2)
LSMOP6	92	3	300	1.9190e+0 (1.09e+0) -	**7.8852e-1** (**4.76e-2**) +	3.7217e+1 (1.32e+1) -	1.3339e+2 (2.77e+2) =	2.4975e+2 (6.13e+2) -	1.4535e+0 (1.49e+0) =	3.4664e+0 (1.37e+0) -	5.0621e+0 (2.29e+0) -	1.4491e+0 (1.86e-1) -	1.7880e+0 (6.54e-1) -	1.1397e+0 (1.99e-1)
LSMOP7	91	3	300	1.0389e+0 (4.72e-2) -	9.2500e-1 (1.05e-2) =	9.0621e-1 (8.48e-2) =	**8.9821e-1** (**5.69e-2**) =	1.2156e+0 (1.53e-1) -	1.5122e+0 (3.70e-2) -	1.5059e+0 (2.35e-2) -	1.9365e+1 (5.57e+0) -	1.4624e+0 (2.65e-1) -	1.2069e+0 (5.76e-2) -	9.0631e-1 (4.31e-2) -
LSMOP8	92	3	300	9.3013e-2 (1.03e-2) -	2.4541e-1 (5.36e-2) -	7.9888e-1 (9.53e-2) -	1.6965e-1 (3.78e-2) -	3.5385e-1 (2.30e-2) -	1.5647e-1 (6.38e-2) -	4.6994e-1 (6.02e-2) -	3.3747e-1 (3.94e-3) -	5.6254e-1 (3.30e-1) -	6.2308e-1 (1.47e-2) -	**1.3579e-2** (**1.23e-2**) -
LSMOP9	92	3	300	5.8893e-1 (2.44e-1) +	5.8387e-1 (8.0e-3) +	1.1570e+0 (2.30e-1) =	1.1429e+0 (3.18e-1) +	2.3751e-0 (3.12e-1) -	2.8701e-0 (4.88e-1) -	3.5314e-0 (1.85e-1) -	2.9149e-0 (6.22e-2) -	1.0544e-0 (9.47e-7) +	**4.9008e-1** (**2.90e-2**) +	1.3816e+0 (2.02e-1) -
+/=/-			1/19/0	5/11/4	0/18/2	1/16/3	1/19/0	5/12/3	1/19/0	2/18/0	3/14/3	2/17/1

**Table 4:** IGD metric of different methods on 8-objective benchmark problems.

Problem	N	M	D	FDV	LMOEADS	IMMOEAD	DGEA	LERD	MOCGDE	AGEMOEAII	HEA	SGECF	UCLMO	TS-SSA
DTLZ1	162	8	800	2.0217e+2 (3.40e+1) -	1.2890e+3 (9.54e+2) -	1.4401e+4 (7.74e+2) -	2.7737e+3 (7.40e+2) -	2.3465e+3 (1.44e+3) -	7.3776e+1 (1.24e+2) -	1.6441e+4 (5.43e+2) -	1.5296e+4 (4.87e+2) -	1.0433e+4 (1.66e+3) -	1.6062e+4 (3.00e+2) -	1.2234e+1 (7.41e+2) -
DTLZ2	162	8	800	6.8972e+0 (5.94e-1) +	1.5423e+1 (9.86e+0) +	3.9960e+1 (2.39e+0) =	5.1501e+0 (7.54e-1) +	1.0981e+1 (5.30e+0) +	5.9780e-1 (1.31e-1) +	4.3311e+1 (1.08e+0) =	1.4303e+1 (3.75e-1) +	1.7332e+2 (3.02e+0) -	2.0004e+0 (1.36e-1) +	2.9393e+1 (8.64e+0) +
DTLZ3	162	8	800	7.6784e+2 (7.99e+1) -	2.5695e+3 (2.19e+3) -	5.8388e+4 (1.58e+3) -	7.1714e+3 (3.91e+3) -	5.2551e+3 (3.68e+3) -	4.7612e+2 (5.25e+2) -	6.9678e+4 (9.70e+2) -	4.1717e+4 (3.13e+3) -	3.3298e+4 (1.05e+4) -	6.8076e+4 (5.42e+2) -	3.2990e+2 (3.05e+3) -
DTLZ4	162	8	800	5.6351e+0 (1.32e+0) +	2.3361e+1 (1.71e+1) =	3.8549e+1 (3.74e+0) -	7.9919e+0 (7.55e-1) +	8.2720e+0 (1.66e+0) +	7.7774e-1 (8.42e-2) +	3.4985e+1 (1.78e+0) -	1.5327e+1 (1.44e+0) =	1.0059e+2 (8.02e-1) -	1.9498e+0 (2.53e-2) -	1.5805e+1 (7.29e+0) -
DTLZ5	162	8	800	6.8237e+0 (9.19e-1) +	2.7270e+1 (1.37e+1) +	3.8003e+1 (3.46e+0) +	5.5877e+0 (9.61e-1) +	1.2093e+1 (2.78e+0) +	2.0947e-1 (1.66e-1) +	5.0650e+1 (1.10e+0) =	2.0260e+1 (8.87e-1) +	1.7972e+2 (1.98e+0) -	1.2183e+0 (2.82e-1) +	3.5074e+1 (1.17e+1) +
DTLZ6	162	8	800	4.0304e+2 (4.26e+1) -	9.6986e+1 (2.50e+1) -	6.8844e+2 (4.42e+0) -	3.2676e+2 (8.86e+1) -	2.0022e+2 (3.46e+1) -	5.6468e-2 (7.36e-2) +	7.0023e+2 (3.56e+0) -	6.5799e+2 (3.32e+0) -	3.1477e+2 (2.11e+2) -	7.1735e+2 (2.12e+0) -	4.7104e+1 (2.59e+1) -
DTLZ7	162	8	800	2.4648e+1 (2.30e+0) -	8.2655e-1 (1.62e-2) -	2.5492e+1 (7.03e-1) -	6.4199e+0 (3.66e+0) =	9.7167e+0 (7.33e-1) -	1.1689e+1 (7.35e+0) -	2.5738e+1 (1.30e+0) -	1.1366e+1 (3.92e-1) -	1.0463e+0 (9.71e-2) -	1.7827e+1 (3.61e-1) -	5.3260e-1 (0.00e+0) -
IDTLZ1	162	8	800	2.1372e+3 (6.43e+2) -	1.1887e+1 (1.48e+1) -	7.2454e+4 (2.41e+3) -	1.7096e+4 (9.70e+3) -	2.2739e+4 (2.26e+3) -	3.0125e+3 (2.85e+3) -	8.5314e+4 (1.13e+3) -	1.6283e+4 (2.84e+2) -	2.5051e+4 (9.29e+1) -	5.6315e+4 (5.29e+3) -	1.1132e+0 (1.86e+0)
IDTLZ2	162	8	800	1.4961e+1 (3.47e+0) =	1.9076e+0 (6.75e-1) =	9.2352e+1 (6.18e+0) -	1.2160e+1 (9.33e+0) =	1.9840e+1 (6.85e+0) =	4.6548e-1 (5.83e-2) +	7.8185e+1 (2.41e+0) -	1.6889e+1 (8.38e-1) -	2.2261e+2 (1.67e+1) -	8.2160e-1 (4.79e-2) +	6.3842e+0 (1.16e+1)
SDTLZ1	162	8	800	9.4856e+2 (1.89e+2) +	9.5120e+3 (1.73e+3) -	4.9314e+5 (1.52e+5) -	8.3454e+3 (1.83e+3) +	3.4713e+4 (3.73e+4) -	2.8263e+4 (4.35e+4) -	3.9788e+4 (5.34e+3) -	3.3842e+4 (8.09e+2) -	2.5353e+4 (2.44e+3) -	1.6025e+4 (1.56e+3) -	9.8047e+1 (1.40e+2)
SDTLZ2	162	8	800	1.6122e+2 (9.63e+1) -	8.8846e+1 (2.56e+1) -	8.5355e+2 (3.17e+2) -	1.1326e+2 (2.89e+1) -	1.3475e+2 (3.17e+1) -	1.7619e+1 (7.10e+0) -	7.0731e+1 (4.26e+0) -	3.9213e+1 (3.15e+0) -	2.6420e+2 (1.01e+2) -	4.1377e+1 (7.92e-1) -	8.3055e+0 (5.35e+0)
LSMOP1	162	8	800	1.3947e+0 (3.20e-1) -	9.7991e-1 (3.68e-2) -	8.2907e+0 (7.17e-1) -	9.9536e-1 (5.69e-2) =	1.8365e+0 (9.84e-1) -	7.2343e-1 (1.85e+0) -	6.5436e+0 (7.81e-1) -	5.1219e+0 (9.50e-1) -	1.0183e+0 (0.00e+0) -	3.9932e-1 (1.62e-2) +	9.7354e-1 (7.42e-3)
LSMOP2	162	8	800	2.2605e-1 (2.21e-3) =	2.5250e-1 (8.24e-3) -	3.9051e-1 (3.75e-2) -	2.3713e-1 (1.36e-2) =	3.3501e-1 (1.28e-2) -	4.8027e-1 (1.02e-1) -	2.8304e-1 (9.88e-3) -	2.4723e-1 (1.61e-3) =	3.0191e-1 (3.79e-3) -	2.1047e-1 (3.40e-3) -	2.5055e-1 (2.31e-3)
LSMOP3	162	8	800	8.6372e+0 (7.14e-1) -	1.5251e+0 (4.29e-1) =	1.1956e+1 (1.24e+1) -	1.1322e+1 (1.19e+0) -	2.0355e+1 (8.28e+0) -	9.8004e+2 (2.25e+3) -	1.5927e+2 (4.30e+2) -	5.7893e+2 (3.24e+2) -	1.8349e+0 (9.0e-1) =	3.1266e+0 (6.89e-2) -	1.6827e+0 (3.23e-1)
LSMOP4	162	8	800	2.5653e-1 (3.08e-3)+	2.8034e-1 (6.67e-3)+	4.1779e-1 (1.97e-2)-	2.7490e-1 (2.00e-2)+	3.8680e-1 (9.99e-2)-	4.8594e-1 (3.79e-2)-	3.2951e-1 (6.44e-3)-	3.003e-1 (2.10e-3)=	3.8225e-1 (9.96e-3)-	2.4068e-1 (3.64e-3)+	3.1678e-1 (1.03e-3)
LSMOP5	162	8	800	1.4094e+0 (7.35e-2)-	1.2575e+0 (1.14e-1)	9.2004e+0 (1.14e+0)-	1.2410e+0 (2.13e-1)-	3.2147e+0 (1.45e+0)-	5.9808e+0 (3.20e+0)-	6.1801e+0 (1.74e+0)-	4.8439e+0 (4.24e-1)-	1.2139e+0 (2.36e-5)	8.3932e-1 (1.18e-2)-	4.0928e-1 (1.03e-2)
LSMOP6	162	8	800	1.2635e+0 (1.35e-2)-	1.7253e+0 (2.47e-1)	1.1956e+1 (1.89e+1)-	1.2390e+1 (2.93e-2)-	1.6708e+0 (6.43e-2)-	1.7836e+3 (4.37e+3)-	1.7523e+0 (3.54e-2)-	2.8473e+1 (2.06e+1)-	1.8658e+0 (7.62e-2)-	1.4176e+0 (7.71e-2)-	1.0694e+0 (1.05e-1)
LSMOP7	162	8	800	3.0564e+1 (8.24e+1)-	2.5565e+0 (8.32e-1)	3.0135e+3 (1.47e+3)-	3.2598e+0 (1.16e+0)-	1.7164e+3 (1.78e+3)-	1.8110e+1 (3.60e+1)-	1.3866e+3 (1.12e+3)-	2.2770e+2 (2.27e+2)-	1.8858e+0 (6.58e-2) =	1.7469e+0 (5.81e-2) =	1.2649e+0 (1.33e-1)
LSMOP8	162	8	800	1.4566e+0 (1.14e-1)-	1.2188e+0 (1.08e-2)	4.5213e+0 (6.80e-1)-	1.0011e+0 (1.77e-1)-	1.8207e+0 (8.84e-1)-	2.0413e+0 (1.58e+0)-	2.2776e+0 (4.54e-1)-	1.2908e+0 (6.74e-2)-	1.1833e+0 (6.14e-2)	8.3109e-1 (5.45e-3)-	4.1199e-1 (5.32e-3)
LSMOP9	162	8	800	3.5702e+2 (2.98e+1)	2.2049e+0 (9.80e-1)=	3.3083e+2 (2.45e+1)-	2.2027e+2 (1.23e+1)-	2.3873e+2 (6.42e+1)-	9.8180e+1 (3.01e+1)-	4.4979e+2 (3.29e+1)-	7.6413e+1 (1.74e+0)-	1.1938e+0 (9.75e-2)+	1.5838e+1 (1.18e+0)-	2.4677e+0 (1.80e+0)
+/=/=			5/13/2	3/10/7	1/18/1	5/11/4	3/15/2	5/15/0	0/18/2	2/15/3	1/17/2	7/12/1

**Table 5:** IGD metric of different methods on 10-objective benchmark problems.

Problem	N	M	D	FDV	LMOEADS	IMMOEAD	DGEA	LERD	MOCGDE	AGEMOEAII	HEA	SGECF	UCLMO	TS-SSA
DTLZ1	276	10	1000	2.4085e+2 (3.36e+1)-	2.5035e+3 (2.14e+3)-	1.7424e+4 (1.05e+3)-	3.3259e+3 (1.43e+3)-	2.7212e+3 (1.01e+3)-	2.4332e+1 (3.07e+1)-	1.9028e+4 (5.48e+2)-	2.0503e+4 (5.17e+2)-	1.2510e+4 (2.27e+3)-	1.8892e+4 (6.25e+2)-	5.3107e+0 (2.26e+3)
DTLZ2	276	10	1000	7.4543e+0 (1.09e+0)	2.0291e+1 (6.3e+1) +	4.4164e+1 (3.3e+0) +	5.0837e+0 (4.47e-1)	9.0708e+0 (4.12e+0)	7.8180e-1 (6.67e-2) +	6.9846e+1 (8.17e-1) =	1.2609e+1 (3.6e+0) +	1.2766e+2 (5.04e-1) -	2.7793e+0 (2.57e-1) +	6.6139e+1 (1.64e+1)
DTLZ3	276	10	1000	8.5432e+2 (1.01e-2) -	3.8004e+3 (4.60e+3) -	6.9971e+4 (2.45e+3) -	7.3874e+3 (3.67e+3) -	4.4751e+3 (3.23e+3) -	1.0278e+2 (1.07e+2) -	9.4628e+4 (6.46e+2) -	5.7260e+4 (2.39e+4) -	2.6279e+4 (2.82e+3) -	8.9875e+4 (1.10e+3) -	1.6806e+1 (4.32e+3)
DTLZ4	276	10	1000	4.1847e+0 (2.90e-1)	3.1400e+1 (7.6e+1) =	5.6075e+1 (5.0e+0) -	1.0089e+1 (8.9e+0) -	1.0453e+1 (2.66e+0) +	8.8517e-1 (6.73e-2) +	6.2136e+1 (4.6e+0) -	1.3039e+1 (3.67e+0) +	8.0343e+1 (2.69e-0) -	3.8476e+0 (9.81e-2) +	4.0039e+1 (4.08e+0)
DTLZ5	276	10	1000	7.2499e+0 (7.15e-1)	5.8536e+1 (8.3e+1)	4.9776e+1 (1.14e+0) +	5.7753e+0 (5.01e-1)	1.6495e+1 (4.39e+0) +	2.8758e-1 (2.8e-1) +	7.0719e+1 (1.21e+0) =	2.0415e+1 (1.08e+1) +	1.9084e+2 (5.81e-1) -	1.6859e+0 (1.19e-1) +	6.5355e+1 (1.15e+0)
DTLZ6	276	10	1000	4.6579e+2 (3.04e+1)	1.9036e+2 (5.2e+1) -	8.4649e+2 (7.08e+0) -	4.8327e+2 (1.37e+1) -	2.1814e+2 (3.80e+1) -	2.1469e-2 (5.3e-2) +	8.288e+2 (1.54e+0) -	7.9549e+2 (6.35e+1) -	1.7814e+2 (5.59e+1) =	8.9195e+2 (3.67e+0) -	5.9270e+1 (3.59e-1)
DTLZ7	276	10	1000	2.9725e+4 (3.2e+0) -	1.1063e+0 (8.3e-3) +	3.1618e+1 (5.10e-1) -	4.5355e+0 (2.03e+0) +	1.4469e+1 (6.83e-1) -	2.0094e+1 (7.30e+0) -	3.8478e+1 (1.77e+0) -	1.1628e+1 (2.58e+0) -	1.2149e+0 (3.76e-2) +	2.3669e+1 (9.44e-1) -	6.5321e+0 (0.00e+0)
IDTLZ1	276	10	1000	2.8952e+3 (7.52e+2)-	2.7908e+1 (2.90e+1)-	1.0519e+5 (2.03e+3)-	1.6780e+4 (1.06e+4)-	3.0070e+4 (4.11e+3)-	5.6956e+3 (4.84e+3)-	1.3285e+5 (1.60e+3)-	2.4283e+4 (2.01e+3)-	3.6111e+4 (2.38e+3)-	9.0745e+4 (1.85e+3)-	**7.2279e-1 (4.34e-1)**
IDTLZ2	276	10	1000	2.0652e+1 (3.82e+0)	2.5304e+0 (1.12e+0)	1.3466e+2 (4.70e+0)-	1.7636e+1 (4.5e+1)	3.1783e+1 (1.05e+1)	**6.0100e-1 (6.0e-2)+**	1.5071e+2 (3.06e-0)	1.2107e+1 (8.4e+1)+	2.1484e+2 (1.69e+1)	1.3050e+0 (9.02e-2)+	2.2708e+1 (2.62e+1)
SDTLZ1	276	10	1000	1.2968e+3 (1.99e+2)=	1.8312e+4 (6.80e+3)	2.5414e+6 (7.06e+5)	1.1317e+4 (1.07e+3)-	2.4794e+5 (1.19e+5)-	1.5774e+5 (2.13e+5)-	2.6213e+5 (1.09e+5)-	6.3655e+5 (1.19e+6)-	4.6836e+4 (1.95e+4)-	2.4707e+4 (4.77e+3)-	**1.2349e+3 (4.62e+2)**
SDTLZ2	276	10	1000	3.8956e+2 (1.96e+2)	2.0926e+2 (4.25e+1)	2.5401e+3 (1.e+2)	2.9598e+2 (1.92e+2)	7.3513e+2 (2.23e+3)	**7.2191e+1 (5.6e+1)+**	3.2046e+2 (4.3e+1)	1.2566e+2 (9.5e+1)	4.3588e+2 (1.96e+2)	1.5043e+2 (6.50e-1)	1.8362e+2 (9.46e+0)
LSMOP1	276	10	1000	9.9593e-1 (2.50e-2)-	1.4865e+0 (1.20e+0)	7.6549e+0 (5.70e-1)	**9.7144e-1 (5.03e-2)**	2.3575e+0 (9.22e-1)	8.6510e+0 (2.45e+0)	7.9797e+0 (3.27e-1)	4.1263e+0 (1.90e+0)	1.0247e+0 (0.00e+0)	3.7571e-1 (1.37e-2)	9.8775e-1 (7.14e-3)
LSMOP2	276	10	1000	2.3099e-1 (9.6e-4)	2.9937e-1 (3.2e-2)	4.2459e-1 (7.4e-2)	2.4434e-1 (9.57e-3)	3.7533e-1 (3.90e-2)	4.7394e-1 (6.29e-2)	2.6737e-1 (5.86e-3)	4.5416e-1 (4.01e-1)	2.9982e-1 (1.97e-3)	2.3113e-1 (2.59e-3)	**1.6663e-1 (2.84e-3)**
LSMOP3	276	10	1000	8.0181e+0 (3.14e-1)	2.3835e+0 (6.54e-1)	4.2497e+2 (6.16e+2)	1.0058e+1 (5.70e+0)	1.8489e+1 (8.66e+0)	3.0099e+3 (3.89e+3)	2.7060e+1 (9.0e+0)	1.9906e+2 (4.0e+2)	1.9186e+0 (5.85e-5)	2.8924e+0 (9.51e-2)	**1.7783e+0 (3.39e-1)**
LSMOP4	276	10	1000	2.5425e-1 (3.37e-3)	3.2263e-1 (3.78e-2)	4.3326e-1 (2.61e-2)	2.8122e-1 (1.30e-2)	3.8717e-1 (1.65e-2)	4.5676e-1 (5.29e-2)	3.0042e-1 (2.24e-3)	4.2524e-1 (2.62e-1)	3.4890e-1 (4.95e-3)	2.4859e-1 (3.05e-3)	**2.1198e-1 (3.28e-3)**
LSMOP5	276	10	1000	1.7180e+0 (6.09e-2)	1.5963e+0 (6.08e-1)	6.5077e+0 (1.67e+0)	1.3375e+0 (2.81e-1)	3.1840e+0 (1.10e+0)	5.3987e+0 (5.26e+0)	1.0121e+1 (1.21e+0)	5.2651e+0 (9.62e-1)	6.4718e+0 (6.27e+0)	8.9245e-1 (6.79e-3)	**4.9980e-1 (2.93e-2)**
LSMOP6	276	10	1000	1.2616e+0 (1.07e-2)	1.5136e+0 (5.24e-2)	6.8266e+0 (9.82e+0)	1.2565e+0 (1.66e-2)	1.4602e+0 (4.61e-2)	8.7241e+2 (1.59e+3)	1.5201e+0 (7.81e-3)	3.1581e+1 (5.35e+1)	3.9533e+2 (7.87e+2)	1.2012e+0 (2.42e-2)	**1.1650e+0 (1.07e-1)**
LSMOP7	276	10	1000	4.6254e+1 (3.30e+1)	1.3896e+1 (1.67e+1)	2.5500e+3 (9.75e+2)	1.1653e+1 (1.57e+1)	2.1360e+3 (1.24e+3)	2.8814e+2 (6.81e+2)	6.7038e+3 (1.53e+3)	5.3258e+2 (2.53e+2)	1.9845e+0 (1.02e-2)	2.0581e+0 (6.12e-2)	**1.3032e+0 (1.79e-1)**
LSMOP8	276	10	1000	1.7281e-0 (6.66e-2)	2.8750e+0 (2.30e+0)	3.6339e+0 (9.95e-1)	1.3822e-0 (4.93e-1)	1.7191e+0 (5.55e-1)	2.0665e+0 (7.85e-1)	5.6355e+0 (6.26e-1)	1.5828e+0 (9.34e-2)	1.1306e+0 (2.04e-1)	8.1520e-1 (7.20e-3)	**4.8814e-1 (3.41e-3)**
LSMOP9	276	10	1000	4.7229e+2 (9.59e+1)	2.0714e+0 (4.35e-1)	4.2386e+2 (4.53e-1)	3.0992e+2 (2.05e+1)	4.6202e+2 (5.53e+1)	2.9467e+2 (4.74e+1)	9.8381e+2 (1.04e+1)	1.1248e+2 (8.23e+1)	**1.5139e+0 (1.13e-1)**	4.5569e+1 (4.01e+0)	2.6772e+0 (2.32e+0)
+ ∕ − ∕ =			3/12/5	2/11/7	2/18/0	4/12/4	3/16/1	6/14/0	0/18/2	5/13/2	1/17/1	6/12/2

**Table 6:** IGD metric of different methods on 15-objective benchmark problems.

Problem	N	M	D	FDV	LMOEADS	IMMOEAD	DGEA	LERD	MOCGDE	AGEMOEAII	HEA	SGECF	UCLMO	TS-SSA
DTLZ1	420	15	1500	3.8502e+2 (5.05e+1)-	1.5957e+3 (2.05e+3)-	2.4345e+4 (1.17e+3)-	4.3569e+3 (1.53e+3)-	4.1207e+3 (2.23e+3)-	8.1401e+2 (1.85e+3)-	2.6431e+4 (1.87e+3)-	2.5589e+4 (8.90e+3)-	2.1628e+4 (1.08e+3)-	1.6591e+4 (2.47e+3)-	**2.9280e+2 (2.88e+3)**
DTLZ2	420	15	1500	1.3632e+1 (2.23e+0)+	3.5338e+1 (3.58e+1)+	8.1752e+1 (5.93e+0)=	9.7237e+0 (1.19e+0)+	2.1823e+1 (7.71e+0)+	**9.6845e-1 (5.73e-2)+**	1.0726e+2 (1.52e+0)=	2.5986e+1 (5.33e+0)+	2.9019e+2 (1.10e+2)-	1.6989e+0 (1.02e+1)+	9.0987e+1 (2.44e+1)
DTLZ3	420	15	1500	**1.5586e+3 (2.36e+2)+**	3.1103e+4 (1.52e+4)=	1.0926e+5 (4.43e+3)-	1.1593e+4 (5.05e+3)=	1.3226e+4 (7.27e+3)=	1.8796e+3 (1.63e+3)+	1.4505e+5 (1.84e+3)-	6.7964e+4 (1.63e+4)-	7.8418e+4 (4.20e+4)-	1.3690e+5 (1.51e+3)-	1.6548e+4 (9.15e+3)
DTLZ4	420	15	1500	6.7900e+0 (1.22e+0)+	2.0186e+1 (2.01e+1)+	8.5080e+1 (5.37e+0)-	1.4008e+1 (2.29e+0)+	1.1051e+1 (2.75e+0)+	**1.0894e+0 (1.52e-1)+**	8.9724e+1 (4.03e+0)-	2.0554e+1 (5.16e-1)+	1.2809e+2 (3.08e+0)-	3.0727e+0 (1.81e+1)+	5.5657e+1 (9.28e+0)
DTLZ5	420	15	1500	1.2945e+1 (7.42e+1)+	9.7961e+1 (2.03e+1)=	7.7967e+1 (4.18e+0)=	6.9269e+0 (3.50e+0)+	1.7382e+1 (6.77e+0)+	**3.2697e-1 (1.24e-1)+**	1.0598e+2 (2.67e+0)+	6.1554e+1 (2.86e+1)+	3.4062e+2 (3.09e+0)-	1.2613e+0 (1.10e+1)+	8.7668e+1 (1.88e+1)
DTLZ6	420	15	1500	6.8953e+2 (9.00e+1)-	2.4573e+2 (1.16e+2)-	1.2554e+3 (2.86e+1)-	4.9805e+2 (1.90e+2)-	4.2120e+2 (8.66e+1)-	**2.5179e-2 (1.75e-2)+**	1.3204e+3 (2.26e+0)-	1.2338e+3 (8.37e+1)-	7.9416e+2 (2.16e+2)-	1.3445e+3 (5.50e+0)-	5.5741e+1 (1.46e-1)
DTLZ7	420	15	1500	5.0727e+1 (4.21e+0)-	2.0335e+0 (1.63e-1)-	5.3491e+1 (5.92e-1)-	1.5325e+1 (4.82e+0)-	2.3173e+1 (2.32e+0)-	4.5342e+1 (5.72e+0)-	5.9692e+1 (1.41e+0)-	3.0649e+1 (4.42e+0)-	2.5027e+0 (1.15e-1)-	4.4094e+1 (8.44e-1)-	**1.2024e+0 (1.05e-1)**
IDTLZ1	420	15	1500	6.5204e+3 (1.13e+3)-	4.4050e+0 (3.33e+0)-	2.0065e+5 (6.51e+3)-	4.8837e+4 (2.26e+4)-	7.4833e+4 (3.00e+4)-	2.0775e+4 (1.13e+4)-	2.5560e+5 (1.10e+3)-	5.8100e+4 (3.85e+3)-	6.9211e+4 (4.71e+2)-	2.3108e+5 (2.05e+3)-	**4.3097e-1 (2.73e-1)**
IDTLZ2	420	15	1500	4.8820e+1 (2.5e+1)+	1.7654e+0 (8.51e-1)+	2.9504e+2 (1.67e+1)-	4.6656e+1 (4.00e+1)=	3.1304e+1 (7.80e+0)=	**8.5191e-1 (3.14e-2)+**	2.9420e+2 (3.69e+0)-	5.3141e+1 (6.76e+0)-	5.8184e+2 (1.90e+2)-	3.9597e+0 (1.64e+1)+	4.9604e+1 (2.89e+1)
SDTLZ1	420	15	1500	5.8504e+3 (1.70e+3)-	1.3851e+5 (1.65e+5)-	1.2271e+8 (1.91e+7)-	1.8308e+4 (4.59e+2)-	1.7953e+7 (0)-	**8.5191e-1 (3.14e-2)+**	2.9420e+2 (3.69e+0)-	5.3141e+1 (6.76e+0)-	5.8184e+2 (1.90e+2)-	3.9597e+0 (1.64e+1)+	4.9604e+1 (2.89e+1)
SDTLZ2	420	15	1500	1.4167e+4 (1.22e+4)-	3.0569e+3 (3.60e+2)=	1.4171e+5 (4.87e+4)-	1.4810e+4 (1.77e+4)=	5.0311e+3 (3.27e+3)-	3.4596e+3 (1.67e+3)-	5.9218e+3 (1.80e+3)-	3.1140e+3 (4.81e+2)=	4.8494e+3 (1.20e+3)-	3.5614e+3 (5.36e+1)-	2.9204e+3 (5.62e+2)
LSMOP1	240	15	1500	9.1333e-1 (5.20e+2)+	2.1310e+0 (1.93e+0)=	7.3450e+0 (8.20e-1)-	9.1027e-1 (6.12e-2)+	3.6623e+0 (9.72e-1)-	1.0233e+1 (3.98e+0)-	6.6942e+0 (1.13e+0)-	5.3974e+0 (3.67e-1)-	1.0440e+0 (0.00e+0)=	**4.6169e-1 (7.4e-2)+**	1.0182e+0 (1.63e+2)
LSMOP2	420	15	1500	2.6321e-1 (2.04e-3)-	3.3874e-1 (5.95e-2)-	5.1254e-1 (3.49e-2)-	2.8863e-1 (1.38e-2)-	4.2949e-1 (3.76e-2)-	5.4858e-1 (1.96e-2)-	4.0470e-1 (1.93e-2)-	4.0674e-1 (2.51e-1)-	3.7073e-1 (5.20e-3)-	2.5749e-1 (2.89e=)	**2.5082e-1 (2.70e-3)**
LSMOP3	420	15	1500	6.1475e+0 (7.11e-1)-	8.744e+0 (8.03e+0)=	3.182e+1 (2.2e+1)-	3.6749e+0 (2.5e+0)=	2.2711e+1 (0.9e+1)-	1.2330e+2 (1.25e-1)-	1.8761e+1 (4.9e-1)-	2.1998e+1 (1.10e+0)-	7.6006e+4 (1.52e+5)-	1.3068e+1 (1.66e+1)-	1.0785e+0 (6.61e-2)
LSMOP4	420	15	1500	2.8540e-1 (5.03e-3)-	3.3615e-1 (5.96e-2)-	5.597e-1 (2.6e-2)-	3.0520e-1 (1.20e-2)-	4.2180e-1 (1.95e-2)-	7.986e-1 (4.40e-2)-	4.3154e-1 (2.17e-2)-	4.022e-1 (64e-1)-	4.223e-1 (47e-2)-	2.6562e-1 (5.42e-3)-	**2.1921e-1 (6.52e-3)**
LSMOP5	420	15	1500	1.5292e+0 (4.32e-2)-	1.3284e+0 (6.39e-2)-	4.4545e+0 (1.19e+0)-	1.1941e+0 (1.46e-1)-	4.9975e+0 (2.0e+0)-	9.1155e+0 (3.53e+0)-	5.4132e+0 (1.02e+0)-	2.4549e+0 (3.03e-1)-	7.3455e+0 (1.21e+1)-	1.0317e+0 (1.08e-2)-	**6.6728e-1 (6.54e-2)**
LSMOP6	420	15	1500	6.9440e+0 (2.32e+0)-	5.4844e+0 (3.18e+0)-	1.4909e+3 (1.12e+3)-	3.1978e+0 (3.10e+0)-	3.0713e+3 (2.57e+3)-	2.8213e+2 (6.64e+2)-	3.8383e+3 (2.28e+3)-	1.2529e+3 (6.37e+2)-	1.9909e+0 (1.11e-2)-	2.1972e+0 (5.94e-2)-	**1.9189e+0 (3.45e-1)**
LSMOP7	420	15	1500	1.4080e+0 (1.75e-2)-	1.5834e+0 (2.74e-1)-	1.6059e+0 (5.99e-2)-	**1.3759e+0 (1.86e-2)**	1.8322e+0 (3.17e-1)-	1.8414e+0 (2.08e-2)-	1.7193e+0 (2.13e-2)-	1.4129e+3 (2.72e+3)-	2.0495e+0 (1.96e-3)-	1.5557e+0 (1.35e-2)-	1.4359e+0 (2.79e-2)
LSMOP8	420	15	1500	1.1379e+0 (4.86e-2)-	1.2574e+0 (5.03e-2)-	1.2597e+0 (9.23e-3)-	1.1569e+0 (3.46e-2)-	1.2361e+0 (7.54e-2)-	1.2561e+0 (5.04e-2)-	1.3117e+0 (1.22e-2)-	1.3081e+0 (6.97e-3)-	6.5631e+0 (9.23e+0)-	1.0987e+0 (1.03e-2)-	**7.9071e-1 (1.72e-1)**
LSMOP9	420	15	1500	1.3759e+1 (2.0e+2)-	**8.5300e+0 (2.28e+0)**	1.1435e+3 (1.15e+1)-	9.0822e+2 (5.63e+1)-	1.6414e+3 (6.33e+2)-	7.5848e+2 (2.41e+2)-	2.5390e+3 (1.19e+2)-	9.3831e+2 (4.48e+2)-	1.6057e+1 (8.57e+0)-	2.9212e+2 (2.54e+1)-	1.0668e+1 (3.22e+0)
+/-/=			5/13/2	3/11/6	0/18/2	4/10/6	3/15/2	6/13/1	0/18/2	3/16/1	0/18/2	5/14/1

**Table 7:** IGD metric of different methods on 20-objective benchmark problems.

Problem	N	M	D	FDV	LMOEADS	IMMOEAD	DGEA	LERD	MOCGDE	AGEMOEAII	HEA	SGECF	UCLMO	TS-SSA
DTLZ1	652	20	2000	5.2878e+2 (5.27e+1)+	2.7187e+4 (3.08e+3)-	3.6914e+4 (2.21e+3)-	9.6496e+3 (3.30e+3)=	3.7934e+3 (1.55e+3)+	3.1054e+3 (2.19e+3)+	3.1572e+4 (2.95e+3)-	2.5621e+4 (9.3e+2)-	1.7805e+4 (1.01e+4)=	1.2750e+4 (8.69e+2)=	1.2134e+4 (3.46e+3)-
DTLZ2	652	20	2000	1.5652e+1 (1.78e+0)+	1.8693e+2 (1.49e+1)-	1.4361e+2 (2.32e+0)-	1.2977e+1 (2.37e+0)+	3.7546e+1 (1.08e+1)+	1.0987e+0 (3.41e-2)	1.5519e+2 (1.22e+0)	6.1829e+1 (9.35e+0)	4.3771e+2 (8.99e+0)	2.2030e+0 (2.63e-1)+	1.2607e+2 (1.63e+1)
DTLZ3	652	20	2000	2.0956e+3 (3.43e+2)+	6.7645e+4 (2.88e+4)=	1.8989e+5 (7.86e+2)-	2.3695e+4 (8.36e+3)+	1.3768e+4 (8.38e+3)+	7.0280e+3 (4.40e+3)+	2.0240e+5 (7.61e+2)-	1.4437e+5 (2.96e+4)-	1.3287e+5 (5.07e+3)-	1.8236e+5 (3.53e+3)-	5.0842e+4 (1.92e+3)
DTLZ4	652	20	2000	7.7156e+0 (7.47e-1)+	7.0372e+1 (6.45e+1)=	1.4117e-2 (5.41e+0)-	2.5671e+1 (4.03e+0)+	1.5585e+1 (2.58e+0)+	1.0619e+0 (9.48e-2)+	1.4778e+2 (4.22e+0)-	3.0614e+1 (3.78e+0)+	1.6715e+2 (4.38e+0)-	3.6024e+0 (2.48e-1)+	9.7359e+1 (3.6e+1)
DTLZ5	652	20	2000	1.8444e+1 (2.67e+0)+	1.5868e+2 (5.86e+1)=	1.4402e+2 (2.54e+0)=	1.1426e+1 (1.18e+0)+	3.9592e+1 (1.30e+1)+	5.1493e-1 (1.75e+1)+	1.5464e+2 (1.36e+0)=	9.4310e+1 (4.60e+1)+	4.3767e+2 (6.11e+0)-	1.7692e+0 (1.18e-1)-	1.4120e+2 (4.37e+1)
DTLZ6	652	20	2000	9.3164e+2 (1.93e+2)-	6.2737e+2 (1.25e+2)-	1.7598e+3 (1.14e+1)-	8.2650e+2 (1.70e+2)-	5.5924e+2 (9.61e+1)-	2.5781e-2 (9.61e-3)+	1.7741e+3 (1.38e+0)-	1.7323e+3 (1.39e+1)-	9.9459e+2 (8.15e+1)-	1.7828e+3 (4.40e+0)-	6.7824e+1 (1.13e+2)
DTLZ7	652	20	2000	6.6266e+1 (1.78e+1)-	1.6307e+1 (5.06e-3)-	8.1267e+1 (1.32e+0)-	2.4798e+1 (9.17e+0)-	3.1052e+1 (6.86e+0)-	7.2823e+1 (7.62e+0)-	8.4745e+1 (1.83e+0)-	5.0014e+1 (2.10e-1)-	2.7684e+1 (1.32e-1)-	5.8525e+1 (4.85e-1)-	1.6300e+1 (3.89e-15)
IDTLZ1	652	20	2000	1.2954e+4 (2.57e+3)-	2.1467e+2 (1.99e+2)-	4.0504e+5 (4.12e+3)-	5.8456e+4 (3.83e+4)-	9.8138e+4 (1.02e+4)-	3.9571e+4 (2.21e+4)-	4.1742e+5 (3.92e+3)-	1.7677e+5 (3.53e+4)-	1.0690e+5 (3.00e+2)-	3.8198e+5 (6.51e+3)-	5.5216e-1 (1.69e+2)
IDTLZ2	652	20	2000	1.1655e+2 (3.19e+1)-	1.5400e+0 (2.55e-1)=	5.6053e+2 (1.53e+1)-	6.7129e+1 (5.75e+1)-	8.7780e+1 (2.39e+1)-	1.0471e+0 (4.70e-2)+	5.5802e+2 (7.61e-1)-	7.0505e+1 (9.26e+0)-	7.1982e+2 (3.64e+1)-	8.3901e+0 (5.28e-1)-	6.7869e+0 (1.18e+2)
SDTLZ1	652	20	2000	3.087e+4 (1.05e+3)+	1.2703e+5 (1.26e+5)-	4.5339e+9 (1.04e+9)-	2.9148e+4 (2.19e+3)=	5.5094e+8 (6.03e+8)-	3.0644e+8 (3.76e+8)-	1.3835e+9 (2.54e+8)-	4.2343e+8 (5.94e+8)-	6.5663e+7 (3.99e+7)-	1.2182e+7 (7.23e+6)-	2.8401e+4 (1.54e+4)
SDTLZ2	652	20	2000	1.3613e+6 (7.12e+5)-	6.7754e+4 (9.81e+3)-	1.1114e+7 (3.19e+6)-	3.7339e+5 (4.49e+5)-	3.7758e+5 (6.82e+5)-	1.1025e+5 (3.54e+4)-	1.7003e+6 (8.62e+5)-	3.9786e+5 (7.36e+5)-	9.7261e+4 (1.93e+4)-	8.0836e+4 (4.43e+3)-	4.2575e+4 (8.36e+3)
LSMOP1	652	20	2000	8.862e-1 (1.45e-1)=	5.1879e+0 (6.31e+0)-	8.7447e+0 (6.65e-1)-	1.0305e+0 (4.74e-2)-	3.7652e+0 (1.73e+0)-	1.2194e+1 (3.40e+0)-	7.8968e+0 (7.89e-1)-	3.0027e+0 (1.36e+0)-	1.0953e+0 (1.54e-3)=	5.2191e-1 (9.43e-3)+	1.0583e+0 (8.13e-4)
LSMOP2	652	20	2000	3.3219e-1 (3.20e-3)-	4.1421e-1 (1.00e-2)-	5.9831e-1 (1.73e-2)-	3.8079e-1 (7.59e-3)-	5.3951e-1 (3.14e-2)-	6.9134e-1 (2.43e-2)-	4.6627e-1 (5.55e-2)-	4.6766e-1 (2.90e-1)=	3.4212e-1 (4.34e-3)-	3.6654e-1 (8.20e-3)-	3.3505e-1 (9.73e-3)
LSMOP3	652	20	2000	6.0835e+0 (1.13e+0)-	1.7979e+1 (2.8e+1)-	1.4937e+2 (1.74e+2)-	7.9730e+0 (2.0e+0)-	1.8385e+1 (3.1e+1)=	1.3845e+2 (1.7e+2)-	3.4639e+1 (3.16e+0)-	1.3400e+4 (9.64e-3)-	1.9901e+0 (1.19e-3)-	1.4680e+0 (1.65e-1)-	1.9701e+0 (2.31e-3)
LSMOP4	652	20	2000	3.4321e-1 (5.83e-3)-	4.5078e-1 (7.74e-3)-	6.3166e-1 (3.16e-2)-	3.9722e-1 (9.55e-3)-	5.9881e-1 (3.27e-2)-	7.6415e-1 (6.32e-2)-	5.1457e-1 (2.43e-2)-	4.7047e-1 (2.55e-1)-	3.6993e-1 (8.06e-3)-	3.6957e-1 (8.09e-3)-	3.0413e-1 (1.10e-2)
LSMOP5	652	20	2000	1.4630e+0 (3.38e-2)-	1.5968e+0 (3.7e-1)-	6.8297e+0 (1.21e+0)-	1.5118e+0 (2.06e-1)-	4.6395e+0 (1.84e+0)-	1.0085e+1 (2.81e-1)-	1.1093e+1 (2.84e+0)-	3.2272e+1 (6.00e+1)-	2.2450e+1 (2.54e+1)-	1.1091e+0 (2.92e-3)-	1.2097e+0 (4.14e-2)
LSMOP6	652	20	2000	1.3530e+0 (8.81e-3)=	1.5050e+0 (1.86e-1)-	1.4105e+1 (9.3e-1)-	1.3482e+0 (1.23e-2)=	1.6725e+0 (9.48e-2)-	4.2160e+2 (1.03e+3)-	1.6799e+0 (9.8e-1)-	2.2684e+3 (4.47e+3)-	1.6532e+0 (1.24e-1)=	1.4539e+0 (3.44e-2)=	1.3531e+0 (7.35e-3)=
LSMOP7	652	20	2000	3.1589e+1 (6.64e+1)-	4.4296e+0 (7.65e-1)-	5.6537e+3 (1.73e+3)-	4.482e+0 (2.08e-2)-	2.6482e+3 (1.26e+3)-	5.0681e+3 (7.4e+3)-	5.2569e+3 (1.17e+3)-	8.9489e+2 (1.02e+2)-	4.9248e+4 (5.69e+4)-	2.4423e+0 (1.37e-1)-	2.0172e+0 (8.12e-2)
LSMOP8	652	20	2000	1.4437e+0 (1.94e+1)=	1.4878e+0 (1.38e+1)=	4.9363e+0 (5.27e-1)-	1.3355e+0 (2.07e-1)-	3.9207e+0 (2.0e+0)-	7.0938e+0 (1.38e+0)-	7.0953e+0 (2.09e+0)-	1.7487e+4 (4.56e+1)-	2.1112e+1 (8.38e+0)-	1.0908e+0 (6.71e-3)-	1.3480e+0 (2.9e-1)-
LSMOP9	652	20	2000	2.5386e+3 (4.02e+2)-	1.6325e+1 (3.14e-2)-	3.0674e+3 (7.66e+1)-	1.7151e+3 (8.16e+1)-	2.8616e+3 (8.94e+1)-	5.4996e+2 (5.91e+1)-	4.7193e+3 (9.65e+1)-	3.1206e+3 (3.98e+3)=	3.4981e+1 (4.77e-1)-	4.7319e+2 (2.47e+1)-	1.3330e+1 (4.62e+0)
+/-/=			6/10/4	0/15/5	0/18/2	4/10/6	5/14/1	7/13/0	0/19/1	3/15/2	4/14/6	5/10/5

From the IGD results in Tables 3, 4, 5, 6, and 7, *TS-SSA* demonstrates significant performance advantages. FDV is a two-stage method that is divided into a fuzzy evolution stage and a precise evolution stage. The fuzzy evolution stage manages convergence for global exploration and the precise evolution phase manages diversity for local exploitation. From the results, *TS-SSA* outperforms FDV on most of the benchmark problems. In the 3-objective benchmark problem, FDV outperforms *TS-SSA* only on the IGD metrics at LSMOP9. Although FDV performs better as the number of objectives increases, it also outperforms *TS-SSA* on only 6 of the 20-objective benchmark problems. The main reason is that although FDV also manages convergence and diversity separately, its two stages are completely separated. The fuzzy evolution stage takes place only in the early search stage and the precise evolution stage takes place only in the late search stage, which results in the inability to enter the precise evolution stage when diversity needs to be improved. Therefore, FDV has a large number of meaningless searches, thus reducing the search efficiency. UCLMO is also a two-stage method. Based on cultural learning, UCLMO proposed the individual selection strategy and the assisted evolution strategy, both of which will alternate during the search process, so that UCLMO outperforms FDV, but still not as good as *TS-SSA*. Because the two stages of UCLMO are carried out in a fixed and alternant manner, which results in that the algorithm does not always enter the right stage at the right time, i.e., it enters the diversity improvement stage when it needs to improve convergence, thus making the search less efficient. In contrast, the two stages of *TS-SSA* adaptively alternate based on population characteristics, which increases the efficiency and accuracy.

LMOEADS is *LSMOEA* and therefore performs well on the 3-objective benchmark problems. However, its performance degrades as the number of objectives increases. On the 3-objective problems, LMOEADS outperforms *TS-SSA* five times on IGD metric, while on the 20-objective problems, LMOEADS does not outperform *TS-SSA* once on IGD metrics. Compared to *MOEAs*, *TS-SSA* has a significant advantage in dealing with *MaOPs*. IMMOEAD is a decomposition-based inverse modeling method, which decomposes the objective space and then constructs an inverse mapping from the objective space to the decision space, and generates solutions in the objective space and then maps them to the decision space. From the results, *TS-SSA* significantly outperforms IMMOEAD. Especially on the LSMOP benchmark problems, *TS-SSA* outperforms IMMOEAD across the board from 3 objectives to 20 objectives. LERD and MOCGDE are also decomposition-based methods. LERD divides the decision variables into convergence and diversity variables according to the analysis of decision variables, and MOCGDE discretizes the covariate gradient to drive individuals to converge and improve diversity on the Pareto front. MOCGDE has better IGD metrics on the DTLZ benchmark problems, but is significantly worse than *TS-SSA* on the LSMOP benchmark problems. To some extent, it shows that when the scale of the decision space is large, methods based on large-scale exploration are superior to those based on decomposition. DGEA is used to improve the optimization of the algorithm from the perspective of generating better offspring. However, generating better offspring is achieved by a pre-selection strategy, and the evolutionary operator still uses GA, which is overall less effective and efficient than SSA. The experimental results also confirm this. HEA enhances selection pressure to select superior offspring through hyper-dominance degree. However, it performs poorly on IDTLZ and SDTLZ benchmark problems with complex shapes of the Pareto front, which is diametrically opposed to *TS-SSA*. *TS-SSA* performs better on complex problems. And this may somewhat indicate that the methods of generating better offspring are better than the methods of enhancing selection pressure for solving complex optimization problems.

**Table 8:** CPU runtime of different methods on 20-objective benchmark problems.

Problem	N	M	D	FDV	LMOEADS	IMMOEAD	DGEA	LERD	MOCGDE	AGEMOEAII	HEA	SGECF	UCLMO	TS-SSA
DTLZ1	652	20	2000	1.5034e+2 (2.68e-2)-	2.7719e+2 (1.05e-1)-	1.8545e+2 (7.57e-2)-	1.2815e+2 (4.66e-2)-	3.6247e+2 (1.75e-2)-	4.0002e+3 (3.7e+0)-	2.0158e+3 (1.24e+0)-	2.4497e+2 (5.88e-2)-	2.4313e+2 (1.27e-2)-	3.533e+2 (5.90e-2)-	8.7301e+1 (5.99e-2)-
DTLZ2	652	20	2000	1.3042e+2 (6.14e-2)-	2.8536e+2 (4.40e-2)-	1.7413e+2 (8.67e-2)-	1.1332e+2 (4.83e-2)-	3.2988e+2 (4.25e-2)-	5.9093e+3 (6.06e+0)-	2.5116e+3 (3.92e+0)-	2.2665e+2 (4.49e-2)-	3.7951e+2 (7.6e-1)-	3.4148e+2 (4.33e-2)-	9.3646e+1 (1.39e-2)-
DTLZ3	652	20	2000	1.3840e+2 (5.17e-2)-	2.8785e+2 (3.34e-2)-	1.8084e+2 (1.10e-1)-	1.2628e+2 (1.27e-2)-	3.9378e+2 (4.94e-2)-	4.1059e+3 (3.32e+0)-	2.0179e+3 (1.49e+0)-	2.3918e+2 (9.67e-2)-	3.4367e+2 (1.17e-1)-	3.5562e+2 (1.70e-1)-	8.8260e+1 (5.81e-2)-
DTLZ4	652	20	2000	1.4473e+2 (1.86e-2)-	2.8133e+2 (2.87e-2)-	1.7648e+2 (1.01e-1)-	1.2693e+2 (3.9e-2)-	3.4501e+2 (4.11e-2)-	7.1773e+3 (1.66e+0)-	1.3897e+3 (1.06e+0)-	1.9858e+2 (1.60e-2)-	3.2112e+3 (3.57e+0)-	3.5284e+2 (8.35e-2)-	8.3980e+1 (8.1e-2)-
DTLZ5	652	20	2000	1.3886e+2 (2.22e-1)-	2.7861e+2 (2.29e-2)-	1.7660e+2 (1.02e-1)-	1.1696e+2 (3.12e-2)-	3.4950e+2 (4.32e-2)-	5.2459e+3 (6.64e+0)-	1.9501e+3 (1.21e+0)-	2.3999e+2 (5.01e-2)-	3.5950e+2 (2.84e-1)-	3.4935e+2 (8.30e-3)-	9.4481e+1 (1.07e-2)-
DTLZ6	652	20	2000	1.3908e+2 (3.7e-2)-	3.0313e+2 (3.96e-2)-	1.9810e+2 (9.67e-2)-	1.2273e+2 (2.9e-2)-	4.8708e+2 (6.58e-1)-	8.5208e+3 (3.31e+0)-	1.9765e+3 (1.29e+0)-	2.5630e+2 (4.1e-2)-	2.9488e+2 (4.64e-1)-	3.8183e+2 (6.78e-2)-	1.0461e+2 (1.10e-2)-
DTLZ7	652	20	2000	3.5203e+2 (5.49e-1)-	2.7192e+2 (3.52e-2)-	1.7704e+2 (3.49e-1)-	1.1472e+2 (5.28e-2)-	4.9913e+2 (7.46e-1)-	2.0950e+3 (2.76e-1)-	2.2427e+3 (1.92e+0)-	2.0784e+2 (1.35e-1)-	9.8860e+2 (1.07e+)-	3.4239e+2 (1.23e-1)-	9.7817e+1 (1.58e-2)-
IDTLZ1	652	20	2000	4.2376e+2 (3.1e+0)-	2.8427e+2 (4.4e-2)-	1.6761e+2 (1.3e-1)-	1.2508e+2 (3.9e-2)-	4.2053e+2 (6.81e-2)-	2.6593e+3 (3.86e-1)-	1.3869e+3 (1.5e+0)-	2.4116e+2 (5.2e-2)-	4.2168e+2 (1.27e+0)-	3.6636e+2 (7.54e-2)-	7.4179e+1 (3.43e-2)-
IDTLZ2	652	20	2000	3.8099e+2 (2.11e+0)-	2.7731e+2 (2.61e-2)-	1.7354e+2 (8.82e-2)-	1.2700e+2 (5.06e-2)-	3.4886e+2 (3.74e-2)-	3.3687e+3 (7.48e-1)-	1.8913e+3 (6.0e+0)-	2.2374e+2 (1.14e-1)-	2.6161e+2 (1.80e-2)-	3.6130e+2 (6.42e-2)-	9.8957e+1 (1.26e-2)
SDTLZ1	652	20	2000	1.3875e+2 (2.50e-2)-	2.9445e+2 (2.98e-2)-	1.6563e+3 (1.24e+0)-	1.2456e+2 (7.48e-2)-	4.2391e+2 (1.40e-1)-	3.0044e+3 (6.98e-1)-	2.2846e+3 (1.73e+0)-	2.5382e+2 (3.95e-2)-	2.7213e+2 (4.63e-2)-	3.7976e+2 (4.05e-2)-	9.1719e+1 (4.54e-2)
SDTLZ2	652	20	2000	1.3498e+2 (4.48e-1)-	2.9015e+2 (4.44e-2)-	4.3746e+2 (9.05e-1)-	1.2276e+2 (1.78e-2)-	3.5046e+2 (2.86e-2)-	2.6853e+3 (2.01e+0)-	2.2914e+3 (3.29e+0)-	2.343e+2 (3.09e-2)-	3.9829e+2 (1.75e-1)-	3.7589e+2 (2.21e-1)-	9.3588e+1 (2.29e-2)
LSMOP1	652	20	2000	1.5137e+2 (5.06e-2)-	3.4025e+2 (5.03e-2)-	1.9130e+2 (1.63e-1)-	1.3460e+2 (3.37e-2)-	9.0814e+2 (1.98e-1)-	2.0674e+4 (6.74e+0)-	2.0061e+3 (2.19e+0)-	2.5522e+2 (5.02e-2)-	1.1410e+2 (3.78e-2)+	5.4438e+2 (9.96e-2)-	9.0583e+1 (1.47e-2)
LSMOP2	652	20	2000	1.6004e+2 (3.22e-2)-	3.6467e+2 (2.97e-2)-	1.9027e+2 (1.02e-1)-	1.3428e+2 (3.03e-2)-	1.0379e+3 (4.41e-1)-	6.5894e+3 (3.81e-1)-	2.1551e+3 (2.90e+0)-	2.6426e+2 (1.95e-2)-	1.7984e+3 (4.25e-1)-	5.8989e+2 (1.96e-1)-	9.5209e+1 (3.94e-2)
LSMOP3	652	20	2000	1.5827e+2 (3.02e‘-2)-	3.3756e+2 (5.05e-2)-	2.0557e+2 (8.11e-2)-	1.3544e+2 (2.44e-2)-	9.6387e+2 (1.85e-1)-	1.7018e+4 (1.81e+3)-	1.9366e+3 (1.01e+0)-	2.2128e+2 (4.06e-2)-	1.2575e+2 (6.87e-3)-	5.3719e+2 (1.84e-1)-	1.0115e+2 (8.64e-2)
LSMOP4	652	20	2000	1.6553e+2 (2.55e-2)	3.4431e+2 (1.15e-2)	2.1560e+2 (9.36e-2)	1.3494e+2 (2.88e-2)	9.2027e+2 (2.92e-2)	8.1055e+3 (3.78e+0)	2.1628e+3 (3.e-1)	2.7470e+2 (2.24e-2)	1.8242e+3 (8.01e-1)	6.1886e+2 (1.81e-1)	9.9879e+1 (6.39e-3)
LSMOP5	652	20	2000	1.4925e+2 (2.73e-1)	3.1587e+2 (3.62e-2)	1.9369e+2 (5.87e-2)	1.2421e+2 (2.13e-2)	8.7137e+2 (2.56e-2)	2.2135e+4 (1.54e+3)	1.5725e+3 (2.03e+0)	2.5751e+2 (2.58e-2)	1.1629e+3 (3.62e-1)	5.6147e+2 (4.71e-2)	8.9621e+1 (1.74e-2)
LSMOP6	652	20	2000	1.4975e+2 (4.45e-2)	3.2916e+2 (2.57e-2)	1.9755e+2 (8.79e-2)	1.2809e+2 (7.57e-3)	1.0049e+3 (1.34e-1)	2.0665e+4 (4.92e+0)	1.0853e+3 (5.66e-1)	2.5695e+2 (2.78e-2)	1.2446e+3 (4.07e-1)	5.952e+2 (1.65e-1)	1.0051e+2 (8.35e-2)
LSMOP7	652	20	2000	1.5046e+2 (1.29e-1)	3.4069e+2 (2.80e-2)	2.0531e+2 (8.33e-2)	1.2668e+2 (1.33e-2)	1.0022e+3 (1.41e-1)	1.5443e+4 (2.09e+3)	9.5605e+2 (2.44e-1)	2.6421e+2 (7.30e-2)	1.2394e+3 (1.01e+0)	6.2681e+2 (1.02e-1)	1.0947e+2 (8.22e-2)
LSMOP8	652	20	2000	1.5085e+2 (8.49e-1)	3.4264e+2 (5.22e-2)	2.1041e+2 (4.79e-2)	1.2553e+2 (2.87e-2)	9.7377e+2 (5.19e-2)	2.6693e+4 (1.79e+3)	1.5700e+3 (2.64e+0)	2.6912e+2 (8.98e-2)	1.3820e+3 (1.27e+0)	6.0156e+2 (3.66e-1)	9.0959e+1 (3.54e-2)
LSMOP9	652	20	2000	8.7559e+2 (1.92e+0)	3.4119e+2 (3.07e-2)	1.9860e+2 (8.92e-2)	1.2403e+2 (3.57e-2)	9.4782e+2 (1.66e-1)	8.3788e+3 (6.05e+0)	2.6650e+3 (1.08e+0)	2.3980e+2 (1.71e-2)	5.1151e+3 (7.92e-1)	6.0296e+2 (2.13e-1)	1.0783e+2 (3.88e-2)
+/-/=			0/20/0	0/20/0	0/20/0	0/20/0	0/20/0	0/20/0	0/20/0	0/20/0	0/20/0	0/20/0

Overall, *TS-SSA*’s performance on the DTLZ problems series deteriorates as the number of targets increases, especially for the 20-objective DTLZ1-5. However, for the more complex and harder to optimize IDTLZ and SDTLZ problem series, *TS-SSA* shows better performance again. The possible reason is that the search space of these two problem types is more complex, and the search strategies of other methods are difficult to achieve a thorough search, so they fall into local optimality. In contrast, *TS-SSA* specializes in search capability and is more adaptable to the huge and complex search space. Similar conclusions can be drawn from the results of the LSMOP problems. In the 3-objective LSMOPs, *TS-SSA* does not show much advantage. However, as the number of objectives increases and the search space becomes more complex, the advantage of *TS-SSA* becomes more obvious. Especially in LSMOPs with more than 10 objectives, *TS-SSA* has a significant advantage over other methods. The results in Table 7 show that none of the methods outperforms *TS-SSA* on the 20-objective LSMOPs. MOCGDE, which performs well on the 20-objective DTLZs, also performs significantly weaker than *TS-SSA* on the LSMOPs.

Table 8 shows the CPU runtime of each method on the 20-objective benchmark problems. According to the results, *TS-SSA* has a significant speed advantage on all problems. Due to the length of the article, the results of CPU running times under other numbers of objectives’ problems are not listed. However, according to the results under different numbers of objectives’ problems, the speed advantage of *TS-SSA* also increases with the number of objectives increasing. From Table 8, we can see that the three decomposition-based methods IMMOEAD, LERD, and MOCGDE are far inferior to *TS-SSA* in terms of time efficiency, especially in LSMOP. In large-scale decision space, the decomposition-based methods need to take a lot of computational resources for analysis and grouping, which results in insufficient computational resources for evolutionary computation and the bad optimization effect. Therefore, in large-scale optimization problems, when the computational resources are limited, the large-scale exploration-based method is superior to the decomposition-based method.

**Fig 3:**
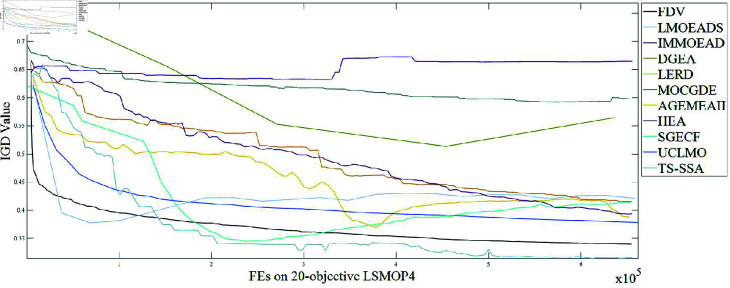
IGD convergence curves of different methods on a 20-objective LSMOP4.

**Fig 4:**
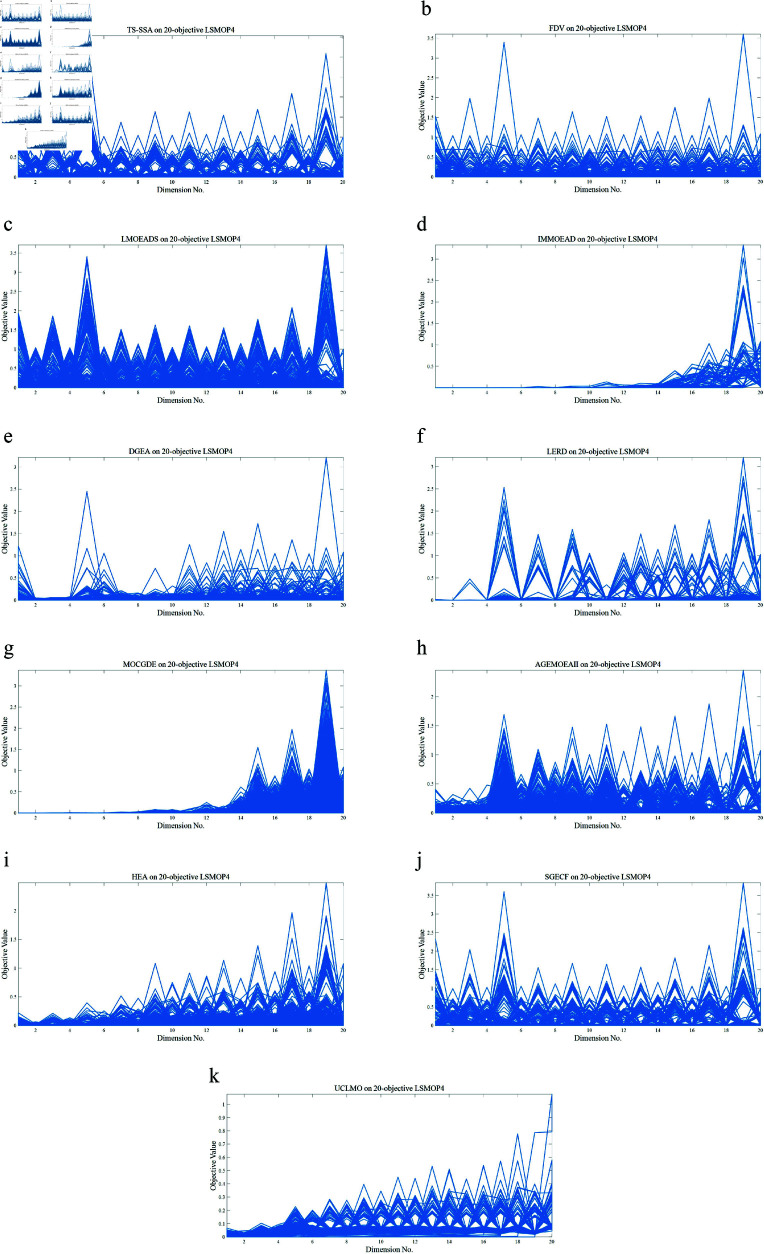
Solution sets of different methods on the 20-objective LSMOP4. (a), (b), (c), (d), (e), (f), (g), (h), (i), (j) and (k) are TS-SSA, FDV, LMOEADS, IMMOEAD, DGEA, LERD, MOCGDE, AGEMOEAII, HEA, SGECF and UCLMO on 20-objective LSMOP4.

Fig 3 shows the IGD convergence curves of different methods on 20-objective LSMOP4. According to the Fig 3, it is shown that *TS-SSA* converges more quickly to the Pareto front than other methods. Fig 4 shows the final solution set of the different methods on 20-objective LSMOP4, where the horizontal coordinate is the objective dimension and the vertical coordinate is the objective value. The solution sets obtained from the *TS-SSA* proposed in this paper show better convergence and diversity compared to other methods.

In summary, *TS-SSA* is able to generate populations with higher convergence and diversity than other state-of-the-art *MOEAs*, which confirms the feasibility of solving the dominance resistance problem existing from the perspective of generating better populations in many-objective spaces. Meanwhile, compared to other *LSMaOEAs*, *TS-SSA* shows a significant advantage in the performance test in the LSMOP suite, which proves the effectiveness of our proposed method in a huge complex search space. And the performance test results in the DTLZ suite show that *TS-SSA* is more advantageous in dealing with problems with complex search spaces, but in regular search spaces, *TS-SSA* performs slightly worse than advanced *MaOEAs*.

**Fig 5:**
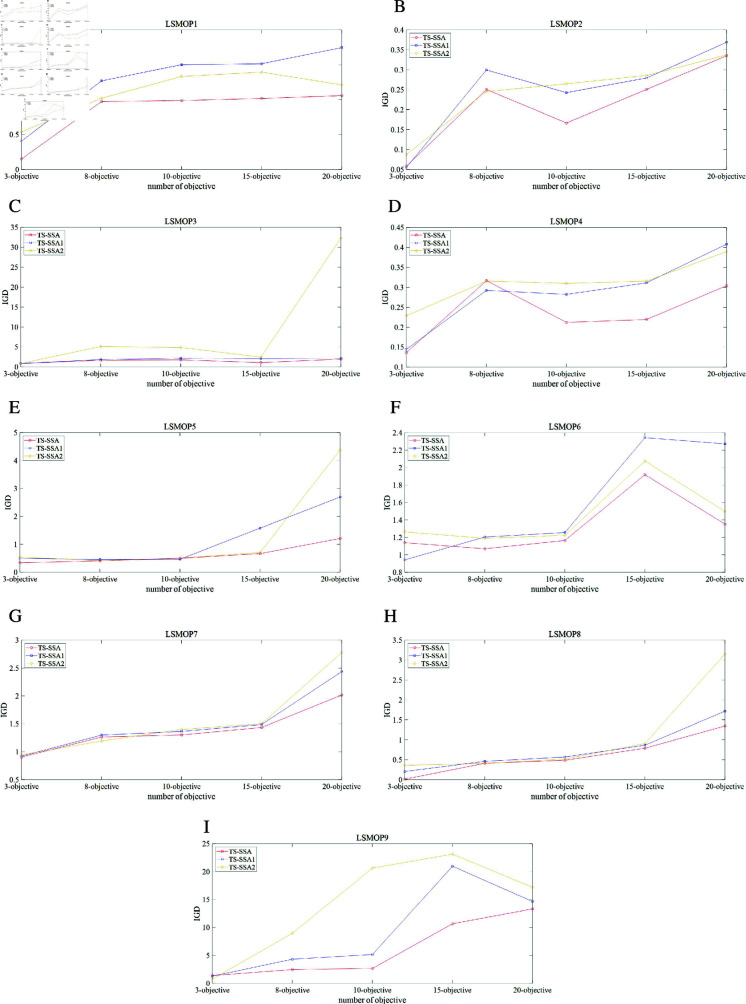
IGD results for 3 methods on 3-20 objective LSMOP. (a), (b), (c), (d), (e), (f), (g), (h) and (i) is IGD results for 3 methods on 3–20 objective from LSMOP1 to LSMOP9.

#### Experiments to demonstrate the validity of the two-stage method.

In this section, we will demonstrate experimentally that it is effective to manage convergence and diversity separately through the two stages. First, we divide TS-SSA into TS-SSA1 and TS-SSA2. In TS-SSA1, only the many-objective sparrow search algorithm is executed, and in TS-SSA2, only the dynamic multi-population search strategy is executed. In order to avoid the situation that there is no follower subpopulation in TS-SSA1 when all individuals converge to the Pareto front, we make some adjustments to TS-SSA1: after all individuals converge to the Pareto front, 40% of the individuals in the population are randomly selected as discoverers, and the rest of the individuals are selected as followers. Fig 5 shows the IGD results of the three methods on the LSMOP with 3, 8, 10, 15, and 20 objectives. According to the IGD results in Fig 3, TS-SSA shows significant advantages over TS-SSA1 and TS-SSA2 in most cases. The two methods TS-SSA1 and TS-SSA2 have their own strengths and weaknesses. TS-SSA1 outperforms TS-SSA2 on LSMOP3, 5, 7, 8, and 9, while TS-SSA2 outperforms TS-SSA1 on LSMOP1, 2, 4, and 6. However, both are not as effective as TS-SSA, which demonstrates the effectiveness of managing convergence and diversity separately through the two stages.

### Application in automatic test scenarios generation

In this section, we apply TS-SSA to a real-world case to examine its optimization effect. Automatic test scenarios generation is a large-scale many-objective optimization problem because test scenarios are intended to cover many test objectives and have many decision nodes [68]. The next part of this section will describe the many-objective automatic test scenarios generation based on UML activity diagrams.

#### UML activity diagrams preprocessing.

**Fig 6:**
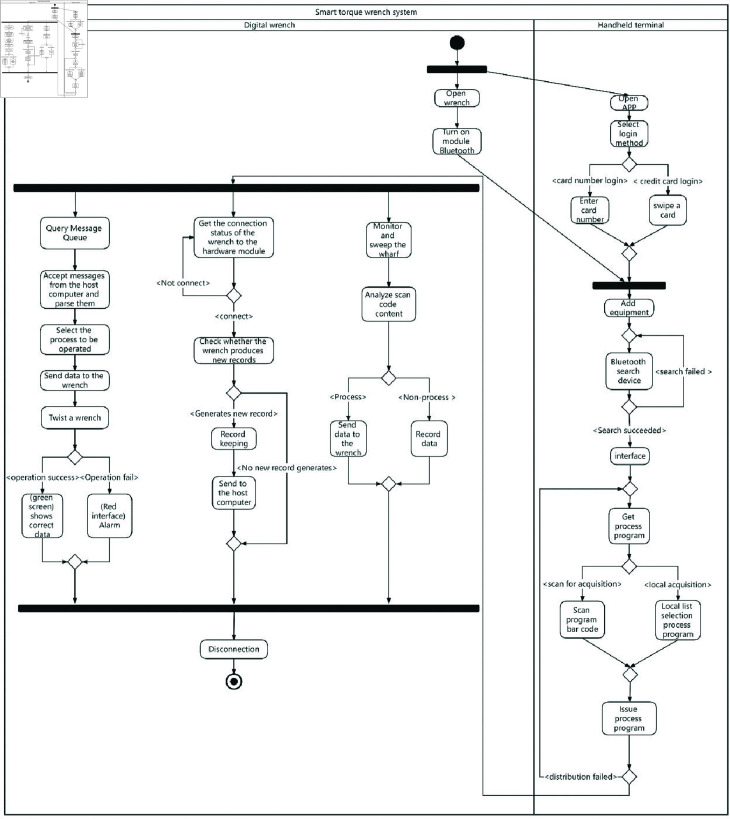
The UML Activity Diagram for Smart Wrench Torque System.

Our previous study successfully automated the generation of test scenarios in UML activity diagrams [69]. However, this study generates test scenarios from a single-objective perspective. The final result shows that there are still many different execution paths that are not covered. The main problem is that generating test scenarios from a single-objective perspective leads to easy omission of some test scenarios because the objectives considered are not sufficient. Especially when there are concurrent activities in the UML activity diagram, the concurrent activities will generate a large number of different execution paths, and it is difficult for the single-objective algorithm to adequately generate test scenarios. Therefore, this time we will generate test scenarios from a many-objective perspective to reduce missed test scenarios. Fig 6 shows the UML activity diagram for the real case used in our previous study, and the exact preprocessing process can be found in our previous study and will not be repeated here.

#### Code design of the solution.

Automatic test scenarios generation from UML activity diagrams is essentially a path generation task on the diagram, which is a discrete problem. In a control flow diagram, paths will fork at decision nodes. Decision nodes are generally branch nodes and loop nodes. Therefore, in this paper, each decision node is treated as a bit, and the value range of the bit is related to the number of branches of the decision node. For example, the value range of a two-branch node is 0 and 1, and the value range of a three-branch node is 0, 1, and 2. A loop node only needs to perform a loop once to cover the cyclic path. So, in this paper, we set the loop to be performed only 0 or 1 time, and the value domain of the loop node is 0 and 1. The code length of the solution is positively related to the number of decision nodes in the UML activity diagrams. In a complete UML activity diagram for a large system, it often contains hundreds or thousands of decision nodes, so the size of the decision variables is huge, and it is a large-scale optimization problem. In the first stage of TS-SSA, the solution space of SSA is in continuous space, so in this paper, a mapping function is designed to map the solution to discrete space. The mapping function is shown in :


Xi,jt+1= { ‖lj⋅ sin ⁡ Xit+1+Xi,jt⋅ tanh ⁡  (2⋅ttmax)‖if i∈SubPopdiscoverer ‖lj⋅ sin ⁡ Xi,jt+1+(Xrd,it−Xrf,it)⋅ tanh ⁡  (2⋅ttmax)‖otherwise (19)

*where* $X_{ij}$ is the j-th position of the i-th solution, $l_{j}$ is the width of the value domain of the j-th position, *t* is the current number of iterations, $X_{rdj}$ is the j-th position of the random individual in the discoverer subpopulation, $X_{rfj}$ is the j-th position of the random individual in the follower subpopulation, and *t_max_* is the maximum of the number of iterations. The discoverers, as the Pareto optimal individuals, will only discretely map with reference to its own previous generation. And the influence of the previous generation will gradually increase as the number of iterations increases. This is because when the search enters the later stage, the position of the individuals are determined. The followers will be close to the discoverers and at the same time away from the other followers to perform the discrete mapping, which ensure the convergence of the solution. It will also increase the influence of the previous generation in the later stages of the search.

The search operators in the second stage of TS-SSA all have discrete versions. Therefore, this paper will directly replace with their discrete versions instead of discrete mapping.

#### Objective function design.

The purpose of automatic uncovered test scenarios generation is to generate a set of test scenarios that cover as many test objectives as possible. Therefore, the set of test scenarios should cover as many nodes and paths as possible. The first minimization objective is to minimize the number of uncovered nodes as shown in :


$$\begin{array}{c} f_{1}(x)=\underset{node\in x}{\sum }w_{node} \end{array}$$ (20)

*where* *x* is a single test scenario, *node* is the node covered by the test scenario *x*, and *w* is the weight of the nodes. The initial weight of each node is 0, and when any test scenario covers the node, its weight is increased by 1. We will maintain a set that includes all nodes. During each round of iteration, the weights of all nodes are first initialized, and then all individuals of the population are traversed to update weight values of each node. Individuals with lower summed weight values are better. Because they cover those uncovered targets.

The second minimization objective is to minimize the number of uncovered edges as shown in :


$$\begin{array}{c} f_{2}(x)=\underset{edge\in x}{\sum }w_{edge} \end{array}$$ (21)

*where* **edge** is the edge covered by the test scenario **x**, and **w** is the weight of the edges. The initial weight of each edge is 0, and when any test scenario covers the edge, its weight is increased by 1. We will maintain a set that includes all edges. During each round of iteration, the weights of all edges are first initialized, and then all individuals of the population are traversed to update weight values of each edge.

To further improve coverage, the third minimization objective is to minimize similarity. Individuals with lower similarity are better. The similarity metrics used in this paper are the Gower-Legendre (dice) similarity metric and the Sokal-Sneath (anti-dice) similarity metric. The Gower-Legendre (dice) similarity measure is shown in and the Sokal-Sneath (Anti-dice) similarity measure is shown in :


f3(x)= max ⁡ y≠x|x∩y||x∪y|+|x∩y|+0.5⋅(|x∪y|−|x∩y|)f4(x)= max ⁡ y≠x|x∩y|−|x∩y||x∪y|+|x∩y|+2⋅(|x∪y|−|x∩y|)(23) (22)

*where* **x** is the current individual, **y** is an individual other than **x**, | *x* ∩ *y* | denotes the number of nodes covered by both individuals together, and | *x* ∪ *y* | denotes the number of all nodes covered by both individuals. The Gower-Legendre similarity measure and the Sokal-Sneath similarity measure have different weights on the denominator of the formula, and the two are used together to better measure the similarity of individuals.

#### Experimental results and analysis.

The application experiments are still running on the PlatEMO platform. DGEA, MOCGDE, and UCLMO, which performed well in the previous comparison experiments, are selected for comparison algorithms.

The evaluation metrics include Node Coverage, Edge Coverage, and the Number of Test Scenarios. Node coverage is the ratio of nodes covered by the set of test scenarios to the total number of nodes. Edge coverage is the ratio of edges covered by the set of test scenarios to the total number of edges. A higher number of test scenarios means that more test objectives are covered and the algorithm is more effective.

The UML activity diagram used for the experiment is the Smart Wrench Torque activity activity diagram shown in Fig 6. The size of population is set to 100. The maximum number of function evaluations is 10000. The average of ten replicate experiments is taken as the final result.

Table 9 shows the experimental results of the automatic test test scenarios generation. From Table 9, we can see that all algorithms cover all nodes and edges, but the number of test scenarios has a large gap. Due to the existence of concurrent modules, many test scenarios cover the same nodes but with different execution paths, which requires more diverse performance of the algorithms. From the results, TS-SSA produces the highest number of test scenarios and performs better compared to other algorithms in terms of diversity. This also proves the effectiveness of the two-stage search strategy.

**Table 9:** The result of automatic test scenarios generation.

Algorithm	Node Coverage (%)	Edge Coverage (%)	Number of Test Scenarios
DGEA	100	100	68.3
MOCGDE	100	100	23.6
UCLMO	100	100	84.7
TS-SSA	100	100	93.1

## Conclusions and future works

This paper proposes a two-stage method to solve LSMaOPs by managing the convergence and diversity of the population separately. In the first stage, the many-objective sparrow search algorithm is proposed to mainly manage convergence through adaptive population dividing strategy and random bootstrap search strategy, which ensures the fast convergence of the population and avoids the extra computational consumption caused by selecting the optimal non-dominant individual. In the second stage, the dynamic multi-population search strategy is proposed to manage diversity. The dynamic multi-population search strategy dynamically changes the three subpopulation sizes and the main search direction of the population according to the value at risk, thus focusing on global exploration in the early search stage and local exploitation in the later search stage. The multi-population search strategy achieves full exploration of the large-scale decision space by searching in three different directions simultaneously.

In order to validate the performance of the proposed method, we conducted several sets of comparison experiments on DTLZ and LSMOP benchmark problems. According to the results of the comparison experiments, the method proposed in this paper has a greater advantage and competitiveness in solving LSMaOPs, which guarantees higher accuracy while possessing lower computational complexity. Then, this paper designs an experiment to demonstrate the effectiveness of managing convergence and diversity separately in two stages. The experimental results show that managing convergence and diversity separately in two stages improves the accuracy of the method, which proves the effectiveness of the method proposed in this paper. In addition, we apply TS-SSA to a real case. The result shows that TS-SSA outperforms other algorithms on diversity.

Although the method proposed in this paper achieves good results in solving LSMaOPs, there are still some aspects that can be improved: first, in this paper, TS-SSA is not used to solve the many-objective optimization problem with constraints or expensive objective function computation, which is a point that can be further explored in future work. Second, TS-SSA currently performs adaptive stage switching based on the condition of whether all populations are at the Pareto front, which is more absolute and may perform poorly in some cases. In future work, we will look for a better switching method. Third, the many-objective improvement of the biological population intelligence algorithm proposed in this paper has so far only been applied to the sparrow search algorithm. We will try to make similar improvements on other biological population intelligence algorithms and propose a possible generalized improvement method.
